# Supplementary material for: Proteomic Comparison of Acute Myeloid Leukemia Cells and Normal CD34+ Bone Marrow Cells: Studies of Leukemia Cell Differentiation and Regulation of Iron Metabolism/Ferroptosis
Source: Proteomes. 2025 Feb 17;13(1):11. doi: 10.3390/proteomes13010011 (PMC11843884; doi:10.3390/proteomes13010011)
Supplement: Supplementary file 1 [file proteomes-13-00011-s001.zip › proteomes-3311660-supplementary.pdf]

Supplementary Materials

Proteomic Comparison of Acute Myeloid Leukemia Cells and Normal CD34$^+$ Bone Marrow Cells: Studies of Leukemia Cell Differentiation and Regulation of Iron Metabolism/Ferroptosis

Table S1: Proteins showing increased levels in primary AML cells compared with normal CD34$^+$ bone marrow cell; Table S2: Proteins showing increased levels in normal CD34$^+$ bone marrow cells compared with primary AML cell; Table S3: Reactome classification of differentially abundant proteins when comparing 50 primary AML cell populations and CD34$^+$ bone marrow cells derived from eight healthy individuals, an overview of proteins showing at least a median 2-fold increase in primary AML cells; Table S4: Differential expression of protein when comparing 50 primary AML cell populations and CD34$^+$ bone marrow cells derived from eight healthy individuals, an overview of proteins showing at least a median 2-fold increase in normal CD34$^+$ cells compared with AML cells; Table S5: Proteins showing a differential expression when comparing AML cells derived from 50 patients and normal CD34$^+$ bone marrow cells derived from eight healthy individuals; an overview of identified proteins after analysis based on Welch's *t*-test with Benjamini correction and fold-change significance (z-score test); Table S6**:** Proteins showing differential expression when comparing AML cells derived from 50 patients and normal CD34$^+$ bone marrow cells derived from eight healthy individuals; an overview of identified proteins after analysis based on Welch's t test with Benjamini correction and fold-change significance (z-score test); Table S7: Hierarchical clustering analysis of 122 differentially abundant proteins when comparing AML cells and normal CD34$^+$ bone marrow cells; the protein subclassification into two main clusters as shown in Figure 5; Table S8: Clinical and biological characteristics of the 50 AML patients included in the study. Patients are listed according to the clustering analysis presented in Figure 5 (listed from left to right, see the top of the figure) in the article, i.e. the upper part of the table represents the left 22 patients in the cluster analysis and the lower part the right subset of 28 patients; Table S9: Clinical and biological characteristics of 50 AML patients included in the study; a comparison of two patient subsets identified by unsupervised hierarchical clustering analysis (Figure 5). Table S10. Protein phosphorylation of primary human AML cells, the identification of protein phosphorylation sites that (i) are localized on a subset (a total of 38 proteins) of the 121 proteins that showed differential expression (i.e. at least 2-fold difference) when comparing primary AML cells and normal CD34$^+$ bone marrow cells; and (ii) in addition a statistically significant difference when each identified phosphosite was compared between the two patient subsets identified in the unsupervised hierarchical clustering analysis presented in Figure 5 (i.e. 23/28 right versus 18/22 left patients). Table S11. Differentially abundant proteins showing additional differences in protein phosphorylation. Table 12. Proteins involved in the regulation of iron homeostasis/metabolism and/or ferroptosis and showing differential expression when comparing primary AML cells and normal CD34$^+$ bone marrow cells. Table S13. Clinical and biological characteristics of the 50 AML patients included in the study; patient subclassification in an unsupervised hierarchical clustering analysis based on 16 differentially abundant proteins involved in regulation of iron metabolism and/or ferroptosis (Figure 6) (listed from left to right, see the top of the figure).

Table S1. Proteins showing increased levels in primary AML cells compared with normal CD34^{+} bone marrow cells. The table presents the 184 proteins that (i) differed significantly between AML cells and normal CD34^{+} bone marrow cells when using the Welch's t-test with Benjamini-Hochberg correction (i.e., FDR<0.01) for the statistical analysis based on those proteins having at least 70% valid protein levels in both group, and (ii) showing a fold change ratio of >2.0 between the median level for AML cells versus normal CD34^{+} cells (i.e. a 2-fold increase in measured protein level in the AML cells compared with the normal CD34^{+} cells). The table presents from left to right the mean fold change (FC), the corresponding p- and q values from the statistical testing, gene name, and protein name for each of the identified proteins,. The proteins marked with yellow color of the gene name represent those 48 proteins that showed statistical significance also after additional Z-statistics (see the later Table S5). The proteins are listed according to the fold change with high fold change proteins at the top.

Mean FC	p-value	q-value	Gene name	Protein name
40.17	7.40E-08	2.35E-06	MVP	Major vault protein
31.84	9.35E-07	2.12E-05	NCF1, NCF1B, NCF1C	Neutrophil cytosol factor 1; Putative neutrophil cytosol factor 1B; Putative neutrophil cytosol factor 1C
30.65	1.80E-05	2.44E-04	IFI30	Gamma-interferon-inducible lysosomal thiol reductase
28.20	1.58E-11	1.61E-09	PLBD1	Phospholipase B-like 1;Phospholipase B-like 1 chain A;Phospholipase B-like 1 chain B;Phospholipase B-like 1 chain C
28.06	2.83E-16	1.37E-13	HBA1	Hemoglobin subunit alpha
26.83	3.09E-10	1.93E-08	HBB	Hemoglobin subunit beta;LVV-hemorphin-7;Spinorphin
24.52	5.61E-05	6.20E-04	KCTD12	BTB/POZ domain-containing protein KCTD12
22.26	2.31E-05	2.96E-04	PLEC	Plectin
18.14	8.35E-10	4.74E-08	HIST1H1E	Histone H1.4
16.09	7.07E-07	1.67E-05	APOBR	Apolipoprotein B receptor
15.56	8.89E-08	2.76E-06	ITGAX	Integrin alpha-X
14.99	2.40E-05	3.05E-04	SIRPA	Tyrosine-protein phosphatase non-receptor type substrate 1
14.79	1.93E-04	1.72E-03	PBXIP1	Pre-B-cell leukemia transcription factor-interacting protein 1
13.65	2.53E-05	3.19E-04	SYNE3	Nesprin-3
13.46	2.31E-04	1.98E-03	S100A8	Protein S100-A8;Protein S100-A8. N-terminally processed
13.36	9.05E-04	6.14E-03	CA2	Carbonic anhydrase 2
11.96	5.02E-04	3.83E-03	CD97	CD97 antigen;CD97 antigen subunit alpha;CD97 antigen subunit beta
11.75	4.45E-09	1.88E-07	AHNAK	Neuroblast differentiation-associated protein AHNAK
10.70	5.06E-08	1.69E-06	TLR2	Toll-like receptor 2
10.39	3.26E-04	2.67E-03	BPI	Bactericidal permeability-increasing protein
9.91	1.32E-05	1.90E-04	S100A9	Protein S100-A9
9.23	7.72E-05	8.10E-04	PRKCD	Protein kinase C delta type
9.16	6.76E-04	4.87E-03	SULT1A1; SULT1A2	Sulfotransferase 1A1;Sulfotransferase 1A2
9.13	2.04E-04	1.80E-03	TRAF3IP3	TRAF3-interacting JNK-activating modulator
8.34	1.57E-03	9.55E-03	ITPR1	Inositol 1.4.5-trisphosphate receptor type 1
7.98	3.80E-07	1.01E-05	CD180	CD180 antigen
7.87	1.10E-12	1.63E-10	ANXA2; ANXA2P2	Annexin A2;Putative annexin A2-like protein
7.24	6.26E-06	1.03E-04	MNDA	Myeloid cell nuclear differentiation antigen
6.97	1.27E-04	1.23E-03	SH3BP1	SH3 domain-binding protein 1

6.89	3.37E-04	2.75E-03	GNS	N-acetylglucosamine-6-sulfatase
6.76	3.00E-06	5.38E-05	ITGAL	Integrin alpha-L
6.70	3.54E-04	2.86E-03	CAPN2	Calpain-2 catalytic subunit
6.57	7.11E-22	8.88E-19	S100A11	Protein S100-A11;Protein S100-A11. N-terminally processed
6.52	1.23E-03	7.93E-03	GLIPR2	Golgi-associated plant pathogenesis-related protein 1
6.24	2.96E-05	3.65E-04	TSPO	Translocator protein
6.15	6.44E-06	1.05E-04	ATG7	Ubiquitin-like modifier-activating enzyme ATG7
5.99	1.03E-04	1.03E-03	HMBS	Porphobilinogen deaminase
5.95	1.19E-04	1.17E-03	SMAP2	Stromal membrane-associated protein 2
5.95	6.50E-09	2.67E-07	LGALS1	Galectin-1
5.92	1.42E-06	2.94E-05	CBL	E3 ubiquitin-protein ligase CBL
5.91	9.86E-05	9.98E-04	ATP6V0D1	V-type proton ATPase subunit d 1
5.75	1.52E-04	1.42E-03	ATM	Serine-protein kinase ATM
5.55	1.07E-03	7.08E-03	SERPINA3	Alpha-1-antichymotrypsin;Alpha-1-antichymotrypsin His-Pro-less
5.21	5.45E-07	1.37E-05	GRN	Granulins;Acrogranin;Paragranulin;Granulin-1;Granulin-2;Granulin-3;Granulin-4;Granulin-5;Granulin-6;Granulin-7
5.18	8.04E-04	5.55E-03	SCPEP1	Retinoid-inducible serine carboxypeptidase
5.07	2.17E-08	7.96E-07	ITGB2	Integrin beta-2
4.92	3.16E-04	2.59E-03	POR	NADPH--cytochrome P450 reductase
4.78	1.34E-05	1.91E-04	GIMAP1	GTPase IMAP family member 1
4.71	1.33E-03	8.45E-03	DIDO1	Death-inducer obliterator 1
4.61	5.19E-04	3.93E-03	DPYD	Dihydropyrimidine dehydrogenase [NADP(+)]
4.55	1.50E-06	3.08E-05	TOM1	Target of Myb protein 1
4.54	8.20E-04	5.64E-03	NXF1	Nuclear RNA export factor 1
4.53	8.50E-07	1.97E-05	RBP4	Retinol-binding protein 4;Plasma retinol-binding protein(1-182);Plasma retinol-binding protein(1-181);Plasma retinol-binding protein(1-179);Plasma retinol-binding protein(1-176)
4.47	1.77E-05	2.42E-04	PTPRC	Receptor-type tyrosine-protein phosphatase C
4.41	2.67E-05	3.33E-04	MRM1	rRNA methyltransferase 1. mitochondrial
4.37	4.25E-04	3.32E-03	CASP1	Caspase-1;Caspase-1 subunit p20;Caspase-1 subunit p10
4.37	2.00E-04	1.77E-03	MYO1F	Unconventional myosin-If
4.32	1.00E-06	2.23E-05	APAF1	Apoptotic protease-activating factor 1
4.32	2.07E-04	1.81E-03	FBP1	Fructose-1.6-bisphosphatase 1
4.32	1.29E-03	8.26E-03	PDPR	Pyruvate dehydrogenase phosphatase regulatory subunit. mitochondrial
4.30	1.07E-03	7.08E-03	MANBA	Beta-mannosidase
4.23	1.62E-03	9.80E-03	SNX27	Sorting nexin-27
4.22	1.28E-10	8.79E-09	HIST1H2AC; HIST3H2A; HIST1H2AB	Histone H2A type 1-C;Histone H2A type 3;Histone H2A type 1-B/E
4.18	2.41E-07	6.67E-06	LYZ	Lysozyme C
4.18	1.92E-06	3.73E-05	ADD3	Gamma-adducin
4.16	8.41E-04	5.76E-03	BST1	ADP-ribosyl cyclase/cyclic ADP-ribose hydrolase 2
4.10	1.21E-03	7.80E-03	FAM21A	WASH complex subunit FAM21A
4.04	5.96E-06	9.92E-05	NCBP2	Nuclear cap-binding protein subunit 2

4.01	1.31E-03	8.36E-03	CPD	Carboxypeptidase D
4.01	8.32E-26	5.19E-22	ANXA1	Annexin A1
3.96	1.55E-04	1.44E-03	FMNL1	Formin-like protein 1
3.88	1.37E-07	4.07E-06	SAMHD1	Deoxynucleoside triphosphate triphosphohydrolase SAMHD1
3.86	3.65E-05	4.30E-04	ALOX5AP	Arachidonate 5-lipoxygenase-activating protein
3.83	1.35E-03	8.53E-03	AHSP	Alpha-hemoglobin-stabilizing protein
3.78	1.39E-04	1.32E-03	RNF213	E3 ubiquitin-protein ligase RNF213
3.73	1.93E-04	1.72E-03	RASSF2	Ras association domain-containing protein 2
3.69	9.60E-18	8.56E-15	VIM	Vimentin
3.67	6.61E-05	7.16E-04	SQRDL	Sulfide:quinone oxidoreductase. mitochondrial
3.66	1.41E-04	1.34E-03	ITGA5	Integrin alpha-5
3.63	1.62E-03	9.80E-03	CTSA	Lysosomal protective protein
3.58	1.17E-07	3.54E-06	IFI35	Interferon-induced 35 kDa protein
3.56	2.12E-04	1.84E-03	TMEM173	Stimulator of interferon genes protein
3.43	8.61E-04	5.85E-03	PPP3CA	Serine/threonine-protein phosphatase 2B catalytic subunit alpha isoform
3.43	8.24E-05	8.53E-04	LMNA	Prelamin-A/C;Lamin-A/C
3.38	6.81E-08	2.19E-06	ARAP1	Arf-GAP with Rho-GAP domain
3.36	2.67E-04	2.24E-03	HTATIP2	Oxidoreductase HTATIP2
3.32	9.27E-04	6.27E-03	FGD3	FYVE. RhoGEF and PH domain-containing protein 3
3.32	1.79E-06	3.53E-05	RPS6KA4	Ribosomal protein S6 kinase alpha-4
3.28	6.80E-14	1.42E-11	RAB11FIP1	Rab11 family-interacting protein 1
3.24	2.18E-04	1.87E-03	SUCLG1	Succinyl-CoA ligase [ADP/GDP-forming] subunit alpha. mitochondrial
3.21	2.84E-06	5.13E-05	SFXN3	Sideroflexin-3
3.09	5.42E-08	1.79E-06	TOLLIP	Toll-interacting protein
3.04	9.01E-07	2.05E-05	CLK3	Dual specificity protein kinase CLK3
3.04	6.75E-06	1.09E-04	SNX18	Sorting nexin-18
3.04	6.55E-04	4.74E-03	PRKCB	Protein kinase C beta type
3.02	1.53E-03	9.37E-03	COQ5	2-methoxy-6-polyprenyl-1.4-benzoquinol methylase. mitochondrial
3.00	1.03E-03	6.83E-03	LMNA	Prelamin-A/C;Lamin-A/C
2.94	1.84E-04	1.66E-03	IDH3G	Isocitrate dehydrogenase [NAD] subunit gamma. mitochondrial
2.92	1.32E-04	1.27E-03	PLBD2	Putative phospholipase B-like 2;Putative phospholipase B-like 2 32 kDa form;Putative phospholipase B-like 2 45 kDa form
2.90	7.60E-04	5.31E-03	ITGA4	Integrin alpha-4
2.87	9.63E-04	6.46E-03	FDXR	NADPH:adrenodoxin oxidoreductase. mitochondrial
2.87	1.68E-12	2.38E-10	TPP1	Tripeptidyl-peptidase 1
2.86	6.70E-04	4.83E-03	PREX1	Phosphatidylinositol 3.4.5-trisphosphate-dependent Rac exchanger 1 protein
2.84	3.95E-05	4.57E-04	MAN2B1	Lysosomal alpha-mannosidase<br>Lysosomal alpha-mannosidase A peptide;Lysosomal alpha-mannosidase B peptide;Lysosomal alpha-mannosidase C peptide;Lysosomal alpha-mannosidase D peptide;Lysosomal alpha-mannosidase E peptide
2.83	1.24E-04	1.21E-03	LRCH4	Leucine-rich repeat and calponin homology domain-containing protein 4
2.82	7.62E-04	5.32E-03	WIPF1	WAS/WASL-interacting protein family member 1
2.79	9.78E-05	9.92E-04	WDFY1	WD repeat and FYVE domain-containing protein 1

2.78	6.80E-04	4.89E-03	RASAL3	RAS protein activator like-3
2.74	1.25E-04	1.22E-03	RNASE6	Ribonuclease K6
2.69	1.10E-05	1.63E-04	FAM101B	Protein FAM101B
2.69	2.33E-05	2.98E-04	CD55	Complement decay-accelerating factor
2.65	1.32E-04	1.27E-03	PPP1R9B	Neurabin-2
2.63	7.14E-04	5.06E-03	HLA-DMB	HLA class II histocompatibility antigen. DM beta chain
2.63	1.27E-03	8.13E-03	GRK6	G protein-coupled receptor kinase 6
2.63	2.31E-04	1.98E-03	ADPGK	ADP-dependent glucokinase
2.63	1.43E-03	8.86E-03	TXNRD2	Thioredoxin reductase 2. mitochondrial
2.60	1.85E-11	1.84E-09	S100A4	Protein S100-A4
2.58	4.45E-04	3.45E-03	MOB4	MOB-like protein phocein
2.57	6.53E-04	4.74E-03	CPNE2	Copine-2
2.55	4.29E-04	3.34E-03	MPG	DNA-3-methyladenine glycosylase
2.55	2.93E-04	2.41E-03	LOC102288414	
2.55	3.13E-09	1.46E-07	MFSD10	Major facilitator superfamily domain-containing protein 10
2.54	5.71E-04	4.25E-03	RDH14	Retinol dehydrogenase 14
2.53	1.32E-05	1.90E-04	C16orf62	UPF0505 protein C16orf62
2.51	2.33E-09	1.13E-07	MYO1G	Unconventional myosin-Ig;Minor histocompatibility antigen HA-2
2.51	2.99E-05	3.67E-04	CNIH4	Protein cornichon homolog 4
2.50	8.92E-09	3.62E-07	GCA	Grancalcin
2.49	2.50E-04	2.13E-03	SLC25A24	Calcium-binding mitochondrial carrier protein SCaMC-1
2.49	1.08E-03	7.14E-03	PLP2	Proteolipid protein 2
2.48	4.26E-08	1.45E-06	NPEPL1	Probable aminopeptidase NPEPL1
2.47	1.19E-06	2.55E-05	H3F3A	Histone H3.3
2.46	6.89E-04	4.93E-03	GSN	Gelsolin
2.46	4.53E-06	7.78E-05	SP110	Sp110 nuclear body protein
2.45	2.17E-04	1.87E-03	WAS	Wiskott-Aldrich syndrome protein
2.45	1.36E-04	1.30E-03	PAK1	Serine/threonine-protein kinase PAK 1
2.44	7.04E-05	7.50E-04	PRKACA	cAMP-dependent protein kinase catalytic subunit alpha
2.44	9.91E-05	1.00E-03	MAP2K3	Dual specificity mitogen-activated protein kinase kinase 3
2.43	2.34E-05	3.00E-04	HIST2H2AC; HIST2H2AA3	Histone H2A type 2-C;Histone H2A type 2-A
2.42	2.65E-04	2.23E-03	SERPINA1	Alpha-1-antitrypsin;Short peptide from AAT
2.42	3.35E-05	4.03E-04	PLEKHO2	Pleckstrin homology domain-containing family O member 2
2.41	7.77E-06	1.23E-04	COL4A3BP	Collagen type IV alpha-3-binding protein
2.40	7.81E-04	5.41E-03	MORC3	MORC family CW-type zinc finger protein 3
2.39	1.57E-05	2.19E-04	ACSL1	Long-chain-fatty-acid--CoA ligase 1
2.39	1.35E-03	8.50E-03	VPS33A	Vacuolar protein sorting-associated protein 33A
2.37	3.78E-06	6.62E-05	ASAH1	Acid ceramidase;Acid ceramidase subunit alpha;Acid ceramidase subunit beta
2.37	1.19E-10	8.43E-09	CD44	CD44 antigen
2.35	2.67E-06	4.89E-05	HIST1H1C	Histone H1.2

2.35	3.49E-14	7.83E-12	CORO1A	Coronin-1A
2.34	2.97E-07	8.05E-06	ICAM3	Intercellular adhesion molecule 3
2.32	3.65E-04	2.93E-03	CREG1	Protein CREG1
2.31	8.51E-07	1.97E-05	RAB8B	Ras-related protein Rab-8B
2.29	1.66E-04	1.53E-03	RAB27A	Ras-related protein Rab-27A
2.29	3.00E-05	3.67E-04	CAPG	Macrophage-capping protein
2.28	1.26E-06	2.67E-05	GLB1	Beta-galactosidase
2.26	1.15E-06	2.50E-05	EML2	Echinoderm microtubule-associated protein-like 2
2.25	7.54E-04	5.28E-03	ETHE1	Persulfide dioxygenase ETHE1. mitochondrial
2.24	1.34E-03	8.48E-03	WDR82	WD repeat-containing protein 82
2.24	6.94E-04	4.95E-03	RHOG	Rho-related GTP-binding protein RhoG
2.23	3.85E-04	3.07E-03	TMX2	Thioredoxin-related transmembrane protein 2
2.23	2.96E-06	5.32E-05	AGTRAP	Type-1 angiotensin II receptor-associated protein
2.23	9.38E-04	6.33E-03	SH3BGRL3	SH3 domain-binding glutamic acid-rich-like protein 3
2.21	1.35E-03	8.50E-03	RHOT1	Mitochondrial Rho GTPase 1
2.20	3.52E-04	2.86E-03	MAP1A	Microtubule-associated protein 1A;MAP1A heavy chain;MAP1 light chain LC2
2.17	5.18E-07	1.32E-05	CDK13	Cyclin-dependent kinase 13
2.17	4.17E-05	4.79E-04	PICALM	Phosphatidylinositol-binding clathrin assembly protein
2.16	7.62E-08	2.40E-06	ATP6V1G1	V-type proton ATPase subunit G 1
2.16	2.84E-06	5.13E-05	ALDH3A2	Fatty aldehyde dehydrogenase
2.16	7.91E-04	5.46E-03	TRRAP	Transformation/transcription domain-associated protein
2.15	1.26E-03	8.11E-03	ESYT2	Extended synaptotagmin-2
2.14	2.62E-06	4.83E-05	LSM7	U6 snRNA-associated Sm-like protein LSm7
2.14	6.90E-05	7.40E-04	DIAPH2	Protein diaphanous homolog 2
2.13	1.26E-04	1.22E-03	ARL8B	ADP-ribosylation factor-like protein 8B
2.11	1.02E-04	1.03E-03	TOR1A	Torsin-1A
2.10	1.96E-07	5.53E-06	OSTF1	Osteoclast-stimulating factor 1
2.10	8.78E-07	2.01E-05	ARHGAP9	Rho GTPase-activating protein 9
2.10	2.91E-04	2.41E-03	CHMP1B	Charged multivesicular body protein 1b
2.08	6.31E-04	4.61E-03	CHTOP	Chromatin target of PRMT1 protein
2.08	1.03E-08	4.13E-07	EBF2	Transcription factor COE2
2.07	2.22E-08	8.09E-07	MBOAT7	Lysophospholipid acyltransferase 7
2.05	5.33E-04	4.01E-03	GAPVD1	GTPase-activating protein and VPS9 domain-containing protein 1
2.04	2.42E-05	3.08E-04	ATP6V1E1	V-type proton ATPase subunit E 1
2.04	1.09E-06	2.39E-05	CTSB	Cathepsin B;Cathepsin B light chain;Cathepsin B heavy chain
2.02	1.04E-04	1.04E-03	HIST2H2BE	Histone H2B type 2-E
2.02	3.62E-09	1.61E-07	AK2	Adenylate kinase 2. mitochondrial;Adenylate kinase 2. mitochondrial. N-terminally processed

Table S2. Proteins showing increased levels in normal CD34$^+$ bone marrow cells compared with primary AML cells. The table presents the 199 proteins that (i) differed significantly between AML cells and normal CD34$^+$ bone marrow cells when using the Welch's t-test with Benjamini-Hochberg correction (i.e., FDR<0.01) for the statistical analysis based on those proteins having at least 70% valid ratios in both group, and (ii) showing an AML/normal ratio corresponding to <0.5 between the mean level for AML cells and normal CD34$^+$ cells (i.e. corresponding to a 2-fold higher level of measured protein in the normal CD34$^+$ cells compared with the AML cells). The table presents from left to right the mean AML/CD34$^+$ fold change (FC), the corresponding p- and q values from the statistical testing, gene name, and protein name for each of the identified proteins,. The proteins marked with yellow represent those 73 proteins that showed statistical significance also after additional Z-statistics (see Table S5). The proteins are listed according to the lowest AML/normal (i.e. relatively highest level in the normal CD34+ cells) fold change at the top.

Mean FC	p-value	q-value	Gene name	Protein name
5.00E-04	1.15E-13	2.18E-11	TF	Serotransferrin
2.03E-03	2.98E-07	8.05E-06	PRAME	Melanoma antigen preferentially expressed in tumors
2.56E-03	2.48E-06	4.67E-05	HPX	Hemopexin
4.29E-03	2.93E-06	5.29E-05	FN1	Fibronectin;Anastellin;Ugl-Y1;Ugl-Y2;Ugl-Y3
9.09E-03	1.48E-06	3.05E-05	PIK3CB	Phosphatidylinositol 4.5-bisphosphate 3-kinase catalytic subunit beta isoform
9.55E-03	2.10E-05	2.77E-04	DENND5A	DENN domain-containing protein 5A
1.14E-02	1.20E-22	2.49E-19	ALDH1A1	Retinal dehydrogenase 1
2.40E-02	3.51E-14	7.83E-12	PRG2	Bone marrow proteoglycan
2.84E-02	2.43E-06	4.59E-05	APOC3	Apolipoprotein C-III
4.42E-02	2.57E-05	3.22E-04	FGA	Fibrinogen alpha chain
5.39E-02	3.44E-09	1.57E-07	APCS	Serum amyloid P-component
5.60E-02	4.18E-05	4.79E-04	TTN	Titin
7.31E-02	1.34E-03	8.50E-03	SACS	Sacsin
7.70E-02	2.98E-05	3.66E-04	FGG	Fibrinogen gamma chain
7.73E-02	1.39E-04	1.33E-03	FGB	Fibrinogen beta chain
9.21E-02	1.58E-17	1.23E-14	PHGDH	D-3-phosphoglycerate dehydrogenase
9.88E-02	8.78E-15	2.38E-12	IGHA1	Ig alpha-1 chain C region
9.89E-02	1.89E-04	1.69E-03	ITGA2B	Integrin alpha-IIb
1.02E-01	2.72E-04	2.27E-03	GP1BA	Platelet glycoprotein Ib alpha chain
1.18E-01	3.94E-04	3.13E-03	TBC1D15	TBC1 domain family member 15
1.23E-01	3.20E-06	5.68E-05	SCRN1	Secernin-1
1.42E-01	3.43E-09	1.57E-07	FHL1	Four and a half LIM domains protein 1
1.47E-01	4.32E-09	1.83E-07	CDK1	Cyclin-dependent kinase 1
1.48E-01	1.80E-06	3.54E-05	MKI67	Antigen KI-67
1.52E-01	6.52E-05	7.07E-04	EPX	Eosinophil peroxidase
1.54E-01	1.27E-10	8.79E-09	ISYNA1	Inositol-3-phosphate synthase 1
1.56E-01	4.20E-04	3.28E-03	GP1BB	Platelet glycoprotein Ib beta chain
1.69E-01	1.49E-04	1.40E-03	HDAC7	Histone deacetylase 7
1.73E-01	3.37E-10	2.07E-08	AKR1C3	Aldo-keto reductase family 1 member C3
1.73E-01	1.21E-05	1.78E-04	UHRF1	E3 ubiquitin-protein ligase UHRF1
1.76E-01	6.41E-04	4.67E-03	PPP1R10	Serine/threonine-protein phosphatase 1 regulatory subunit 10
1.79E-01	1.17E-06	2.54E-05	MZB1	Marginal zone B- and B1-cell-specific protein
1.87E-01	2.29E-06	4.36E-05	HMGN5	High mobility group nucleosome-binding domain-containing protein 5

1.93E-01	1.23E-06	2.63E-05	DBN1	Drebrin
1.99E-01	4.18E-08	1.43E-06	HSPB1	Heat shock protein beta-1
1.99E-01	5.51E-07	1.38E-05	RRM1	Ribonucleoside-diphosphate reductase large subunit
1.99E-01	7.26E-04	5.13E-03	ROCK2	Rho-associated protein kinase 2
2.13E-01	7.02E-11	5.64E-09	LIG1	DNA ligase 1
2.14E-01	2.53E-05	3.19E-04	HP	Haptoglobin
2.24E-01	1.97E-05	2.63E-04	PRTN3	Myeloblastin
2.28E-01	9.78E-05	9.92E-04	FKBP11	Peptidyl-prolyl cis-trans isomerase FKBP11
2.28E-01	2.15E-04	1.86E-03	TOP2A	DNA topoisomerase 2-alpha
2.30E-01	1.83E-14	4.40E-12	ADH7	Alcohol dehydrogenase class 4 mu/sigma chain
2.31E-01	1.78E-05	2.42E-04	CD9	CD9 antigen
2.38E-01	3.83E-07	1.01E-05	TFRC	Transferrin receptor protein 1
2.41E-01	1.62E-03	9.80E-03	CCNK	Cyclin-K
2.41E-01	6.14E-06	1.02E-04	TMEM123	Porimin
2.52E-01	5.32E-07	1.34E-05	CMBL	Carboxymethylenebutenolidase homolog
2.62E-01	1.33E-05	1.90E-04	CLC	Galectin-10
2.70E-01	9.68E-12	1.10E-09	DNPH1	2-deoxynucleoside 5-phosphate N-hydrolase 1
2.71E-01	9.64E-08	2.98E-06	MSI2	RNA-binding protein Musashi homolog 2
2.71E-01	4.14E-17	2.87E-14	CAD	CAD protein;Glutamine-dependent carbamoyl-phosphate synthase
2.73E-01	1.76E-04	1.61E-03	TRMT61A	tRNA (adenine(58)-N(1))-methyltransferase catalytic subunit TRMT61A
2.85E-01	3.21E-09	1.48E-07	DNMT1	DNA (cytosine-5)-methyltransferase 1
2.87E-01	1.90E-06	3.70E-05	PTGS1	Prostaglandin G/H synthase 1
2.93E-01	3.39E-10	2.07E-08	ATPAF1	ATP synthase mitochondrial F1 complex assembly factor 1
2<E-01	1.52E-13	2.68E-11	SMC4	Structural maintenance of chromosomes protein 4
2.98E-01	3.94E-05	4.57E-04		Ig kappa chain V-III region WOL
3.00E-01	2.85E-16	1.37E-13	MCM2	DNA replication licensing factor MCM2
3.02E-01	5.66E-14	1.22E-11	PTMA	Prothymosin alpha
3.05E-01	2.06E-06	3.96E-05	MAD2L1	Mitotic spindle assembly checkpoint protein MAD2A
3.06E-01	2.24E-05	2.89E-04	CD59	CD59 glycoprotein
3.06E-01	6.93E-05	7.40E-04	TMEM14C	Transmembrane protein 14C
3.09E-01	5.66E-06	9.48E-05	PSMG2	Proteasome assembly chaperone 2
3.09E-01	7.02E-05	7.50E-04	H1F0	Histone H1.0;Histone H1.0. N-terminally processed
3.12E-01	5.59E-10	3.26E-08	FKBP4	Peptidyl-prolyl cis-trans isomerase FKBP4
3.15E-01	6.08E-22	8.88E-19	DUT	Deoxyuridine 5-triphosphate nucleotidohydrolase. mitochondrial
3.19E-01	2.43E-05	3.08E-04	ALDH7A1	Alpha-aminoadipic semialdehyde dehydrogenase
3.24E-01	1.15E-08	4.57E-07	ACY1	Aminoacylase-1
3.24E-01	7.52E-06	1.20E-04	FSCN1	Fascin
3.24E-01	2.65E-11	2.51E-09	STMN1	Stathmin
3.31E-01	4.79E-05	5.37E-04	REXO2	Oligoribonuclease. mitochondrial

3.34E-01	2.18E-10	1.40E-08	LANCL1	LanC-like protein 1
3.34E-01	4.91E-10	2.92E-08	PFAS	Phosphoribosylformylglycinamidine synthase
3.37E-01	3.97E-04	3.14E-03	SELL	L-selectin
3.39E-01	1.36E-05	1.93E-04	NCAPH	Condensin complex subunit 2
3.39E-01	5.77E-09	2.39E-07	GRWD1	Glutamate-rich WD repeat-containing protein 1
3.42E-01	3.49E-05	4.17E-04	GSTM2	Glutathione S-transferase Mu 2
3.42E-01	1.91E-08	7.16E-07	FABP5	Fatty acid-binding protein. epidermal
3.45E-01	1.97E-05	2.63E-04	ACTN1	Alpha-actinin-1
3.45E-01	3.40E-11	3.17E-09	PEBP1	Phosphatidylethanolamine-binding protein 1
3.45E-01	6.91E-05	7.40E-04	HLA-DPA1	HLA class II histocompatibility antigen
3.46E-01	1.32E-09	7.11E-08	MIF	Macrophage migration inhibitory factor
3.46E-01	4.73E-16	1.74E-13	MCM6	DNA replication licensing factor MCM6
3.47E-01	5.37E-08	1.78E-06	GART	Trifunctional purine biosynthetic protein adenosine-3
3.47E-01	4.94E-19	5.14E-16	PCNA	Proliferating cell nuclear antigen
3.49E-01	2.85E-08	1.01E-06	ATXN10	Ataxin-10
3.51E-01	7.60E-17	4.74E-14	MCM3	DNA replication licensing factor MCM3
3.51E-01	4.84E-07	1.24E-05	RAD23B	UV excision repair protein RAD23 homolog B
3.53E-01	6.85E-07	1.63E-05	PRPS2	Ribose-phosphate pyrophosphokinase 2
3.55E-01	4.58E-16	1.74E-13	PAICS	Multifunctional protein ADE2
3.56E-01	6.80E-06	1.09E-04	RCOR3	REST corepressor 3
3.56E-01	7.71E-05	8.10E-04	MSL1	Male-specific lethal 1 homolog
3.57E-01	1.33E-08	5.24E-07	PM20D2	Peptidase M20 domain-containing protein 2
3.60E-01	1.58E-06	3.20E-05	XPO5	Exportin-5
3.62E-01	2.71E-12	3.68E-10	MSH6	DNA mismatch repair protein Msh6
3.63E-01	2.01E-16	1.14E-13	MCM7	DNA replication licensing factor MCM7
3.64E-01	8.10E-16	2.81E-13	MCM4	DNA replication licensing factor MCM4
3.65E-01	1.38E-04	1.32E-03	SMC2	Structural maintenance of chromosomes protein 2
3.67E-01	3.60E-04	2.89E-03	HIST1H2AJ;<br>HIST1H2AH;<br>H2AFJ;<br>HIST1H2AG;<br>HIST1H2AD	Histone H2A type 1-J<br>Histone H2A type 1-H<br>Histone H2A.J<br>Histone H2A type 1<br>Histone H2A type 1-D
3.67E-01	2.09E-08	7.71E-07	CALU	Calumenin
3.68E-01	2.91E-09	1.37E-07	BZW2	Basic leucine zipper and W2 domain-containing protein 2
3.76E-01	3.86E-16	1.63E-13	MCM5	DNA replication licensing factor MCM5
3.76E-01	2.11E-05	2.77E-04	PSMG1	Proteasome assembly chaperone 1
3.77E-01	7.81E-11	6.09E-09	ADH5	Alcohol dehydrogenase class-3
3.78E-01	1.97E-09	1.01E-07	NAA15	N-alpha-acetyltransferase 15
3.79E-01	1.25E-10	8.76E-09	STAT5A	Signal transducer and activator of transcription 5A
3.81E-01	1.65E-11	1.66E-09	CDK6	Cyclin-dependent kinase 6
3.81E-01	8.81E-10	4.92E-08	TTLL12	Tubulin--tyrosine ligase-like protein 12

3.83E-01	1.42E-07	4.18E-06	IPO4	Importin-4
3.86E-01	8.10E-04	5.58E-03	JADE2	Protein Jade-2
3.86E-01	9.02E-10	4.98E-08	AARS	Alanine--tRNA ligase. cytoplasmic
3.87E-01	8.18E-06	1.29E-04	ALDH2	Aldehyde dehydrogenase. mitochondrial
3.88E-01	2.97E-07	8.05E-06	SORD	Sorbitol dehydrogenase
3.88E-01	9.49E-11	7.14E-09	MTHFD1	C-1-tetrahydrofolate synthase. cytoplasmic
3.90E-01	5.65E-06	9.48E-05	STMN2	Stathmin-2
3.93E-01	1.03E-12	1.57E-10	NAP1L1	Nucleosome assembly protein 1-like 1
3.97E-01	2.22E-09	1.09E-07	PAFAH1B3	Platelet-activating factor acetylhydrolase IB subunit gamma
4.00E-01	4.46E-06	7.69E-05	IGLC2;<br>IGLC3;IGLC1	Ig lambda-2 chain C regions;Ig lambda-3 chain C regions;Ig lambda-1 chain C regions
4.01E-01	1.50E-06	3.08E-05	DCTPP1	dCTP pyrophosphatase 1
4.01E-01	1.09E-08	4.38E-07	CSDE1	Cold shock domain-containing protein E1
4.02E-01	3.12E-06	5.56E-05	ACOT7	Cytosolic acyl coenzyme A thioester hydrolase
4.04E-01	1.17E-04	1.15E-03	RIF1	Telomere-associated protein RIF1
4.04E-01	5.85E-07	1.44E-05	OXSR1	Serine/threonine-protein kinase OSR1
4.05E-01	1.54E-13	2.68E-11	NASP	Nuclear autoantigenic sperm protein
4.07E-01	5.49E-06	9.28E-05	HNRNPC	Heterogeneous nuclear ribonucleoproteins C1/C2
4.08E-01	6.82E-04	4.90E-03	HBS1L	HBS1-like protein
4.09E-01	6.50E-12	8.11E-10	YWHAQ	14-3-3 protein theta
4.10E-01	2.35E-05	3.00E-04	HSPB11	Intraflagellar transport protein 25 homolog
4.12E-01	4.83E-15	1.44E-12	MSH2	DNA mismatch repair protein Msh2
4.14E-01	1.84E-08	6.93E-07	NPM3	Nucleoplasmin-3
4.15E-01	4.14E-11	3.64E-09	C4orf27	UPF0609 protein C4orf27
4.17E-01	1.74E-14	4.35E-12	LDHB	L-lactate dehydrogenase B chain
4.20E-01	1.67E-08	6.42E-07	PRDX1	Peroxiredoxin-1
4.20E-01	2.17E-10	1.40E-08	TXNL1	Thioredoxin-like protein 1
4.20E-01	7.06E-04	5.02E-03	PUS7	Pseudouridylate synthase 7 homolog
4.23E-01	6.83E-11	5.61E-09	RANBP1	Ran-specific GTPase-activating protein
4.24E-01	8.45E-04	5.78E-03	SLC27A2	Very long-chain acyl-CoA synthetase
4.25E-01	6.56E-08	2.12E-06	NUDT1	7.8-dihydro-8-oxoguanine triphosphatase
4.25E-01	1.63E-04	1.51E-03	CDK2	Cyclin-dependent kinase 2
4.25E-01	1.53E-06	3.11E-05	AKR1B1	Aldose reductase
4.28E-01	4.56E-05	5.15E-04	HLA-DRA	HLA class II histocompatibility antigen DR alpha chain
4.29E-01	4.11E-04	3.22E-03	CSTB	Cystatin-B
4.29E-01	9.01E-11	6.94E-09	IMPDH2	Inosine-5-monophosphate dehydrogenase 2
4.30E-01	1.51E-03	9.29E-03	HLA-C	HLA class I histocompatibility antigen. Cw-7 alpha chain
4.32E-01	2.73E-04	2.27E-03	ASF1A	Histone chaperone ASF1A
4.33E-01	1.02E-06	2.25E-05	NOC2L	Nucleolar complex protein 2 homolog
4.37E-01	2.11E-05	2.77E-04	RSF1	Remodeling and spacing factor 1
4.38E-01	6.25E-04	4.58E-03	BCR	Breakpoint cluster region protein

4.41E-01	2.71E-09	1.28E-07	POLD3	DNA polymerase delta subunit 3
4.41E-01	1.97E-09	1.01E-07	NACA	Nascent polypeptide-associated complex subunit alpha. muscle-specific form
4.42E-01	5.73E-05	6.31E-04	SH3BGRL	SH3 domain-binding glutamic acid-rich-like protein
4.43E-01	3.21E-23	1.00E-19	EEF1B2	Elongation factor 1-beta
4.46E-01	2.22E-05	2.87E-04	ORC3	Origin recognition complex subunit 3
4.48E-01	7.81E-04	5.41E-03	LZTFL1	Leucine zipper transcription factor-like protein 1
4.48E-01	7.70E-13	1.23E-10	PTPLAD1	Very-long-chain (3R)-3-hydroxyacyl-CoA dehydratase 3
4.50E-01	1.07E-03	7.07E-03	OAS3	2-5-oligoadenylate synthase 3
4.51E-01	1.69E-10	1.12E-08	PABPC4	Polyadenylate-binding protein 4
4.52E-01	2.00E-04	1.77E-03	MACROD1	O-acetyl-ADP-ribose deacetylase MACROD1
4.52E-01	3.56E-04	2.87E-03	TTC27	Tetratricopeptide repeat protein 27
4.53E-01	7.98E-15	2.26E-12	EIF5A;<br>EIF5AL1	Eukaryotic translation initiation factor 5A-1
4.55E-01	5.67E-10	3.28E-08	YARS	Tyrosine--tRNA ligase. cytoplasmic
4.56E-01	1.06E-05	1.58E-04	RHEB	GTP-binding protein Rheb
4.56E-01	2.01E-08	7.48E-07	MAN2C1	Alpha-mannosidase 2C1
4.56E-01	8.16E-07	1.90E-05	SRM	Spermidine synthase
4.60E-01	1.22E-05	1.79E-04	UBR7	Putative E3 ubiquitin-protein ligase UBR7
4.61E-01	3.09E-06	5.52E-05	ACO1	Cytoplasmic aconitate hydratase
4.62E-01	1.52E-03	9.32E-03	DDAH2	N(G).N(G)-dimethylarginine dimethylaminohydrolase 2
4.62E-01	9.10E-06	1.40E-04	MARCKSL1	MARCKS-related protein
4.63E-01	5.39E-04	4.04E-03	KRTCAP2	Keratinocyte-associated protein 2
4.68E-01	3.43E-10	2.08E-08	GMPS	GMP synthase [glutamine-hydrolyzing]
4.69E-01	2.32E-09	1.13E-07	LARS	Leucine--tRNA ligase. cytoplasmic
4.69E-01	5.19E-07	1.32E-05	IPO9	Importin-9
4.71E-01	2.72E-04	2.27E-03	MINA	Bifunctional lysine-specific demethylase<br>and histidyl-hydroxylase MINA
4.71E-01	4.10E-07	1.07E-05	TUBA1C	Tubulin alpha-1C chain
4.74E-01	1.02E-11	1.12E-09	UROD	Uroporphyrinogen decarboxylase
4.74E-01	1.53E-14	3.97E-12	PDCD4	Programmed cell death protein 4
4.75E-01	3.49E-11	3.21E-09	IARS	Isoleucine--tRNA ligase. cytoplasmic
4.76E-01	1.70E-06	3.38E-05	GARS	Glycine--tRNA ligase
4.77E-01	2.15E-05	2.81E-04	CIAPIN1	Anamorsin
4.77E-01	2.18E-05	2.83E-04	NEDD4	E3 ubiquitin-protein ligase NEDD4
4.78E-01	4.01E-09	1.73E-07	TPP2	Tripeptidyl-peptidase 2
4.81E-01	1.04E-03	6.94E-03	SFXN2	Sideroflexin-2
4.81E-01	1.71E-05	2.36E-04	PFDN5	Prefoldin subunit 5
4.81E-01	1.41E-03	8.80E-03	SYNGR1	Synaptogyrin-1
4.81E-01	1.18E-06	2.55E-05	RNASEH2B	Ribonuclease H2 subunit B
4.82E-01	8.74E-07	2.01E-05	CTPS1	CTP synthase 1
4.83E-01	7.30E-04	5.14E-03	TSTD1	Thiosulfate sulfurtransferase/rhodanese-like domain-containing protein 1

4.85E-01	3.55E-06	6.25E-05	CRKL	Crk-like protein
4.85E-01	6.50E-07	1.57E-05	ATIC	Bifunctional purine biosynthesis protein PURH
4.85E-01	2.55E-06	4.76E-05	DNAJC2	DnaJ homolog subfamily C member 2
4.91E-01	3.80E-09	1.65E-07	GEMIN4	Gem-associated protein 4
4.94E-01	4.11E-07	1.07E-05	ADSL	Adenylosuccinate lyase
4.94E-01	6.83E-07	1.63E-05	UQCR10	Cytochrome b-c1 complex subunit 9
4.96E-01	4.19E-06	7.27E-05	TRIM25	E3 ubiquitin/ISG15 ligase TRIM25
4.98E-01	8.58E-14	1.73E-11	PRMT1	Protein arginine N-methyltransferase 1
4.99E-01	2.28E-04	1.95E-03	EEFSEC	Selenocysteine-specific elongation factor
4.99E-01	8.82E-10	4.92E-08	RPLP2	60S acidic ribosomal protein P2
4.99E-01	6.70E-07	1.62E-05	IPO7	Importin-7

Table S3. Reactome classification of differentially abundant proteins when comparing 50 primary AML cell populations and CD34^{+} bone marrow cells derived from eight healthy individuals, an overview of proteins showing at least a median 2-fold increase in primary AML cells ***<u>(see Table 1 for a complete list)</u>***. The figure shows the 79 proteins (left column) included in the 10 top-ranked Reactome terms (see top of the figure). Proteins associated with neutrophil differentiation are marked with yellow, green color indicates intracellular signaling, blue color cellular interactions and purple color regulation of apoptosis. The R-HAS numbers are given together with the Reactome terms. All the 10 terms showed p-values <0.00005.

	Neutrophil degranulation 6798695	Innate immune system 168249	Iimmune system 168265	Adaptive immune system 1280218		Signaling by GTPase-R-HSA 194315	RHO GTPases-activate NADPH oxidases 5668599	Signaling by RHO-and Miro GTPases 9716542		Cell surface interactions at vascular wall 202733	INTEGRIN cell surface interactions 216083S		Apoptotic cleavage of cellular proteins 111465
**ADD3**													
**ALDH3A2**													
**ANXA1**													
**ANXA2**													
**APAF1**													
**ARAP1**													
**ARHGAP9**													
**ASAH1**													
**ATG7**													
**ATP6V1E1**													
**ATP6V1G1**													
**ATP6VOD1**													
**BPI**													
**BST1**													
**CASP1**													
**CBL**													
**CD44**													
**CD55**													
**CD180**													
**CDK13**													
**CPNE2**													
**CREG1**													
**CTSA**													
**CTSB**													
**DIAPH2**													
**FMNL1**													
**GCA**													
**GLB1**													
**GNS**													

GRN										
GSN										
HLA-DMB										
ICAM3										
IFI30										
IFI35										
ITGA4										
ITGA5										
ITGAL										
ITGAX										
ITGB2										
ITPR1										
LMNA										
LYZ										
MAN2B1										
MANBA										
MAP2K3										
MNDA										
NCF1										
OSTF1										
PICALM										
PLEC										
PLEKHO2										
PPP3CA										
PRKCB										
PRKCD										
PRKCB										
PREX1										
PTPRC										
RAB27A										
RHOG										
RHOT1										
RNASE6										
RNF213										
S100A8										
S100A9										
S100A11										
SAMHD1										
SERPINA1										
SERPINA3										
SH3BP1										
SIRPA										
TLR2										
TOLLIP										
TOM1										
WAS										
VIM										
WIPF1										

Table S4. Differential expression of protein when comparing 50 primary AML cell populations and CD34^{+} bone marrow cells derived from eight healthy individuals, an overview of proteins showing at least a median 2-fold increase in normal CD34^{+} cells compared with AML cells ***(see Table 2 for the complete list)***. The figure shows the 79 proteins (left column) included in the 10 top-ranked Reactome terms (see top of the figure). Proteins associated with purine/nucleotide metabolism are marked with yellow, green color indicates DNA function/cell cycle regulation and blue color MAPK signaling. The R-HAS numbers are given together with the Reactome terms. All the 10 terms showed p-values <0.00005.

	Purine ribonucleotide monophosphaye biosynthesis 73817	Metabolism of nucleotides 15869	Nucleotide biosynthesis 8956320	DNA strand elongation 69190	Activation of prereplicative complex 68962	Unwinding of DNA 176974	Activation of ATR in response to replicative stress 176187	Mitotic G1 phase and and /S transition 453279	p130 CAS linkage to MAPK signaling for integrins 372708	GRB2:SOS linkage to MAPK signaling for integrins 354194
**ADSL**	■ (yellow)	■ (yellow)	■ (yellow)							
**ATIC**	■ (yellow)		■ (yellow)							
**IMPDH2**	■ (yellow)		■ (yellow)							
**GMPS**	■ (yellow)	■ (yellow)	■ (yellow)							
**GART**	■ (yellow)	■ (yellow)	■ (yellow)							
**PAICS**	■ (yellow)	■ (yellow)	■ (yellow)							
**PFAS**	■ (yellow)	■ (yellow)	■ (yellow)							
**DUT**		■ (yellow)								
**RRM1**		■ (yellow)								
**NUDT1**		■ (yellow)								
**CTPSA**		■ (yellow)								
**DNPH1**		■ (yellow)								
**ATIC**		■ (yellow)								
**IMPDH2**		■ (yellow)								
**DCTPP1**		■ (yellow)								
**POLD3**				■ (green)						
**PCNA**				■ (green)				■ (green)		
**MCM7**				■ (green)	■ (green)	■ (green)		■ (green)		
**MCM3**				■ (green)	■ (green)	■ (green)		■ (green)		
**MCM4**				■ (green)	■ (green)	■ (green)		■ (green)		
**MCM5**				■ (green)	■ (green)	■ (green)		■ (green)		
**MCM6**				■ (green)	■ (green)	■ (green)		■ (green)		
**ORC3**					■ (green)			■ (green)		
**CDK2**					■ (green)					
**CDK1**								■ (green)		
**CDK6**								■ (green)		
**TOP2A**								■ (green)		
**ORC3**								■ (green)		

**FGB**										
**FGA**										
**FGG**										
**ITGA2B**										
**FN1**										

Table S5. Proteins showing a differential expression when comparing AML cells derived from 50 patients and normal CD34$^+$ bone marrow cells derived from eight healthy individuals; an overview of identified proteins after analysis based on Welch's *t*-test with Benjamini correction and fold-change significance (z-score test). The statistical analysis was based on those proteins having at least 70% valid ratios in both groups. The table presents the 121 proteins that differed significantly between AML cells and normal CD34$^+$ bone marrow cells; red color indicates increased levels in AML cells and blue color increased levels in the normal CD34$^+$ cells. The table presents from left to right the fold change ***ratio of log$_2$ LFQ intensity values***, the p values from the statistical comparisons, the gene name and the protein names for each identified protein. The proteins are ranked according to the ratio of primary AML cells versus CD34$^+$ bone marrow cells derived from healthy individuals.

Median ratio AML/CD34$^+$	p-value	Gene name	Protein names
5.86	0.0002	MVP	Major vault protein
5.80	0.0002	NCF1/1B/1C	Neutrophil cytosol factor 1
5.62	0.0003	KCTD12	BTB/POZ domain-containing protein KCTD12
5.51	0.0003	PLBD1	Phospholipase B-like 1
5.28	0.0005	HBA1	Hemoglobin subunit alpha
5.13	0.0007	HBB	Hemoglobin subunit beta;LVV-hemorphin-7;Spinorphin
5.12	0.0008	IFI30	Gamma-interferon-inducible lysosomal thiol reductase
4.40	0.0029	PBXIP1	Pre-B-cell leukemia transcription factor-interacting protein 1
4.39	0.0029	SIRPA	Tyrosine-protein phosphatase non-receptor type substrate 1
4.33	0.0032	PLEC	Plectin
4.24	0.0038	APOBR	Apolipoprotein B receptor
4.22	0.0039	SYNE3	Nesprin-3
3.99	0.0057	HIST1H1E	Histone H1.4
3.99	0.0058	S100A8	Protein S100-A8;Protein S100-A8, N-terminally processed
3.94	0.0063	PRKCD	Protein kinase C delta type
3.91	0.0065	AHNAK	Neuroblast differentiation-associated protein AHNAK
3.88	0.0068	ITGAX	Integrin alpha-X
3.77	0.0082	TLR2	Toll-like receptor 2
3.72	0.0088	CD97	CD97 antigen subunit alpha/beta
3.61	0.0104	S100A9	Protein S100-A9
3.59	0.0108	BPI	Bactericidal permeability-increasing protein
3.50	0.0124	CD180	CD180 antigen
3.42	0.0139	ITPR1	Inositol 1,4,5-trisphosphate receptor type 1
3.34	0.0156	ATG7	Ubiquitin-like modifier-activating enzyme ATG7
3.33	0.0159	TRAF3IP3	TRAF3-interacting JNK-activating modulator
3.23	0.0181	ITGAL	Integrin alpha-L
3.16	0.0200	DPYD	Dihydropyrimidine dehydrogenase [NADP(+)]
3.16	0.0201	SH3BP1	SH3 domain-binding protein 1
3.13	0.0208	ANXA2/2P2	Annexin A2;Putative annexin A2-like protein
3.07	0.0227	CA2	Carbonic anhydrase 2
3.02	0.0244	MNDA	Myeloid cell nuclear differentiation antigen
2.98	0.0255	CAPN2	Calpain-2 catalytic subunit
2.98	0.0255	SULT1A1/2	Sulfotransferase 1A1;Sulfotransferase 1A2
2.95	0.0265	CBL	E3 ubiquitin-protein ligase CBL
2.92	0.0279	S100A11	Protein S100-A11
2.91	0.0282	GLIPR2	Golgi-associated plant pathogenesis-related protein 1
2.89	0.0288	ATM	Serine-protein kinase ATM
2.89	0.0290	SMAP2	Stromal membrane-associated protein 2
2.86	0.0301	GNS	N-acetylglucosamine-6-sulfatase
2.73	0.0353	GRN	Granulins 1-7

2.70	0.0366	APAF1	Apoptotic protease-activating factor 1
2.64	0.0397	TSPO	Translocator protein
2.63	0.0403	LYZ	Lysozyme C
2.61	0.0410	SCPEP1	Retinoid-inducible serine carboxypeptidase
2.54	0.0446	SERPINA3	Alpha-1-antichymotrypsin
2.54	0.0450	ATP6V0D1	V-type proton ATPase subunit d 1
2.49	0.0476	LGALS1	Galectin-1
2.49	0.0479	PDPR	Pyruvate dehydrogenase phosphatase regulatory subunit, mitochondrial
-1.50	0.0498	TRMT61A	tRNA (adenine(58)-N(1))-methyltransferase catalytic subunit TRMT61A
-1.50	0.0497	FKBP4	Peptidyl-prolyl cis-trans isomerase FKBP4
-1.51	0.0472	PCNA	Proliferating cell nuclear antigen
-1.51	0.0471	CLC	Galectin-10
-1.51	0.0471	RCOR3	REST corepressor 3
-1.52	0.0467	PTMA	Prothymosin alpha
-1.54	0.0431	STMN1	Stathmin
-1.55	0.0423	SMC2	Structural maintenance of chromosomes protein 2
-1.57	0.0397	GRWD1	Glutamate-rich WD repeat-containing protein 1
-1.60	0.0353	DUT	Deoxyuridine 5-triphosphate nucleotidohydrolase, mitochondrial
-1.63	0.0324	TMEM14C	Transmembrane protein 14C
-1.64	0.0313	JADE2	Protein Jade-2
-1.64	0.0311	CAD	CAD protein;Glutamine-dependent carbamoyl-phosphate synthase
-1.67	0.0273	DNPH1	2-deoxynucleoside 5-phosphate N-hydrolase 1
-1.68	0.0270	SMC4	Structural maintenance of chromosomes protein 4
-1.73	0.0227	MAD2L1	Mitotic spindle assembly checkpoint protein MAD2A
-1.74	0.0214	TOP2A	DNA topoisomerase 2-alpha
-1.74	0.0213	PRTN3	Myeloblastin
-1.78	0.0186	CD9	CD9 antigen
-1.82	0.0161	MSI2	RNA-binding protein Musashi homolog 2
-1.83	0.0152	ALDH7A1	Alpha-aminoadipic semialdehyde dehydrogenase
-1.84	0.0149	ACY1	Aminoacylase-1
-1.87	0.0130		Ig kappa chain V-III region
-1.89	0.0123	MSL1	Male-specific lethal 1 homolog
-1.89	0.0120	CMBL	Carboxymethylenebutenolidase homolog
-1.94	0.0097	PSMG2	Proteasome assembly chaperone 2
-1.95	0.0095	ADH7	Alcohol dehydrogenase class 4 mu/sigma chain
-1.95	0.0093	CD59	CD59 glycoprotein
-1.99	0.0081	REXO2	Oligoribonuclease, mitochondrial
-2.00	0.0076	HP	Haptoglobin;Haptoglobin alpha chain;Haptoglobin beta chain
-2.01	0.0074	H1F0	Histone H1.0;Histone H1.0, N-terminally processed
-2.06	0.0059	TFRC	Transferrin receptor protein 1
-2.09	0.0054	HDAC7	Histone deacetylase 7
-2.09	0.0052	MZB1	Marginal zone B- and B1-cell-specific protein
-2.12	0.0046	PPP1R10	Serine/threonine-protein phosphatase 1 regulatory subunit 10
-2.15	0.0040	DBN1	Drebrin
-2.15	0.0040	FKBP11	Peptidyl-prolyl cis-trans isomerase FKBP11
-2.20	0.0032	AKR1C3	Aldo-keto reductase family 1 member C3
-2.24	0.0027	UHRF1	E3 ubiquitin-protein ligase UHRF1
-2.29	0.0021	ISYNA1	Inositol-3-phosphate synthase 1
-2.29	0.0020	HMGN5	High mobility group nucleosome-binding domain-containing protein 5
-2.32	0.0018	HSPB1	Heat shock protein beta-1
-2.37	0.0014	LIG1	DNA ligase 1
-2.38	0.0013	EPX	Eosinophil peroxidase
-2.41	0.0011	RRM1	Ribonucleoside-diphosphate reductase large subunit
-2.45	0.0009	CCNK	Cyclin-K
-2.46	0.0009	ROCK2	Rho-associated protein kinase 2
-2.49	0.0008	TMEM123	Porimin
-2.61	0.0004	MKI67	Antigen KI-67
-2.76	0.0002	CDK1	Cyclin-dependent kinase 1
-2.85	9.63E-05	FHL1	Four and a half LIM domains protein 1

-2.96	4.69E-05	IGHA1	Ig alpha-1 chain C region
-3.00	3.78E-05	GP1BB	Platelet glycoprotein Ib beta chain
-3.14	1.48E-05	ITGA2B	Integrin alpha-IIb heavy chain
-3.14	1.47E-05	GP1BA	Platelet glycoprotein Ib alpha chain;Glycocalicin
-3.45	1.64E-06	SCRN1	Secernin-1
-3.51	1.02E-06	PHGDH	D-3-phosphoglycerate dehydrogenase
-3.61	4.83E-07	TBC1D15	TBC1 domain family member 15
-3.94	3.13E-08	SACS	Sacsin
-4.03	1.44E-08	FGG	Fibrinogen gamma chain
-4.12	6.74E-09	FGB	Fibrinogen beta chain
-4.29	1,34E-09	APCS	Serum amyloid P-component
-4.36	7.33E-10	FGA	Fibrinogen alpha chain
-4.95	1.65E-12	PRG2	Bone marrow proteoglycan
-4.96	1.43E-12	TTN	Titin
-5.12	2.44E-13	APOC3	Apolipoprotein C-III
-6.47	<0.0001	ALDH1A1	Retinal dehydrogenase 1
-7.47	<0.0001	DENND5A	DENN domain-containing protein 5A
-7.54	<0.0001	PIK3CB	Phosphatidylinositol 4,5-bisphosphate 3-kinase catalytic subunit beta isoform
-8.11	<0.0001	FN1	Fibronectin
-9.05	<0.0001	PRAME	Melanoma antigen preferentially expressed in tumors
-9.40	<0.0001	HPX	Hemopexin
-11.19	<0.0001	TF	Serotransferrin

Table S6. Proteins showing differential expression when comparing AML cells derived from 50 patients and normal $CD34^+$ bone marrow cells derived from eight healthy individuals; an overview of identified proteins after analysis based on Welch's t test with Benjamini correction and fold-change significance (z-score test). The statistical analysis was based on those proteins having at least 70% valid ratios in both groups. The table presents those differentially abundant proteins that (i) differed significantly between AML cells and normal $CD34^+$ bone marrow cells; and either (ii) showed increased levels in AML cells and were included in the corresponding Reactome terms (Figure 2A, see also Figure 3) or (iii) were identified in the Volcano analysis (Figure 2B).

GENE NAME	PROTEIN NAME AND FUNCTION	KEY WORDS
	**Neutrophil Degranulation / Innate Immune System**<br>(Increased in primary AML cells)	
SERPINA3	*Serpin family A member 3.* The encoded protein is a member of the serpin family that inhibit serine proteases. This gene is one in a cluster of serpin genes located on the q arm of chromosome 14. Polymorphisms in this protein appear to be tissue specific and influence protease targeting.	Protease inhibition
GRN	*Granulin precursor.* Granulins are secreted, glycosylated peptides that are cleaved from the single 88 kDa precursor progranulin protein. Cleavage of the signal peptide produces mature granulin which can be further cleaved into a variety of active, 6 kDa peptides named granulin A, granulin B, granulin C, etc. Both the peptides and intact granulin protein regulate cell growth. However, different members of the granulin protein family may act as inhibitors, stimulators, or have dual actions on cell growth.	Granulins<br>Growth regulation
APAF1	*Apoptotic peptidase activating factor 1.* This cytoplasmic protein initiates apoptosis. The protein contains a caspase recruitment domain and an ATPase domain. Upon binding cytochrome c and ATP this protein forms an oligomeric apoptosome that binds and cleaves caspase 9 preproprotein and thereby releases its mature, activated form. Activated caspase 9 stimulates the subsequent caspase cascade that commits the cell to apoptosis.	Apoptosis<br>Cytochrome c<br>Caspase 9
NCF1	*Neutrophil cytosolic factor 1.* The protein is a 47 kDa cytosolic subunit of neutrophil NADPH oxidase. It is a multicomponent enzyme that is activated to produce superoxide anion.	NADPH oxidase<br>Neutrophil
ANXA2	*Annexin A2.* This member of the annexin family that play a role in the regulation of cellular growth and in signal transduction pathways. This protein functions as an autocrine factor in osteoclasts, and its expression correlates with resistance to treatment against various cancer forms.	Autocrine regulation<br>Chemoresistance
PRKCD	*Protein kinase C delta.* This member of the protein kinase C family of serine- and threonine-specific protein kinases is activated by diacylglycerol and is both a tumor suppressor and a positive regulator of cell cycle progression. Also, this protein can positively or negatively regulate apoptosis.	Tumor suppressor<br>Cell cycle regulation<br>Apoptosis
ITGAL	*Integrin subunit alpha L.* The encoded integrin alpha L chain that forms a heterodimeric membrane protein with the beta 2 chain (ITGB2); this dimer is also referred to as the lymphocyte function-associated antigen-1 (LFA-1). It plays a central role in leukocyte intercellular adhesion through interactions with its ligands, ICAMs 1-3 (intercellular adhesion molecules 1 through 3), but it can also functions in lymphocyte costimulatory signaling.	Integrin<br>Adhesion<br>Communication
ITGAX	*Integrin subunit alpha x.* The alpha X chain integrin protein combines with the beta 2 chain (ITGB2) to form a leukocyte-specific integrin referred to as inactivated-C3b (iC3b) receptor 4 (CR4). Thiscomplex seems to overlap the properties of the alpha M beta 2 integrin in the adherence of neutrophils and monocytes to stimulated endothelium, and in the phagocytosis of complement coated particles.	Endothelium<br>Adhesion
LYZ	*Lysozyme.* The natural substrate of this protein is the bacterial cell wall peptidoglycan. Lysozyme is an antimicrobial agent that is present in spleen, lung, kidney, white blood cells, plasma, saliva, and tears. The protein has antibacterial activity against a number of bacterial species.	Antibacterial<br>Secreted
GNS	*Glucosamine (N-acetyl)-6-sulfatase.* The encoded lysosomal enzyme is found in all cells and is involved in the catabolism of heparin, heparan sulphate, and keratan sulphate.	Lysosome
SIRPA	*Signal regulatory protein alpha.* The encoded protein is a member of the signal-regulatory-protein (SIRP) family, and also belongs to the immunoglobulin superfamily. SIRP family members are receptor-type transmembrane glycoproteins known to be involved in the negative regulation of receptor tyrosine kinase-coupled signaling processes. This protein can be phosphorylated by tyrosine kinases. The phospho-tyrosine	Surface receptor<br>Intracellular signaling<br>CD47

	residues of this PTP have been shown to recruit SH2 domain containing tyrosine phosphatases (PTP), and serve as substrates of PTPs. This protein was found to participate in signal transduction mediated by various growth factor receptors. CD47 is a ligand for this receptor.	
BPI	*Bactericidal permeability increasing protein,* This gene encodes a lipopolysaccharide binding protein. It is associated with human neutrophil granules and has antimicrobial activity against gram-negative organisms.	Neutrophil granules
MNDA	*Myeloid cell nuclear differentiation antigen.* This myeloid marker is detected only in nuclei of cells of the granulocyte-monocyte lineage. Its mRNAwhich contains an interferon-stimulated response element, and its expression was upregulated in human monocytes exposed to interferon alpha. This protein resembles IFI16 In its pattern of expression and/or regulation, suggesting that these genes participate in blood cell-specific responses to interferons.	Myeloid marker<br>Interferon<br>Granulocyte<br>Monocyte
ATG7	Autophagy related 7. This E1-like activating enzyme is essential for autophagy and cytoplasmic to vacuole transport. It is also thought to modulate p53-dependent cell cycle pathways during prolonged metabolic stress. The protein has been associated with multiple functions, including mitophagy and hematopoietic stem cell maintenance.	Autophagy<br>Mitophagy<br>P53<br>Hematopoietic stem cell
S100A9	*S100 calcium binding protein A9.* The protein is a member of the S100 protein family. S100 proteins are localized in the cytoplasm and/or nucleus of a wide range of cells, and involved in the regulation of cellular processes such as cell cycle progression and differentiation. S100 genes include at least 13 members. This protein may function in the inhibition of casein kinase. This antimicrobial protein exhibits antifungal and antibacterial activity.	Calcium metabolism<br>Calcium<br>Casein kinase<br>Cytoplasm/nucleus<br>Cell cycle<br>Differentiation<br>Antimicrobial
S100A11	*S100 calcium binding protein A11.* The encoded protein is a member of the S100 protein family that are localized in the cytoplasm and/or nucleus This protein possibly functions in motility, invasion, and tubulin polymerization, including cancer metastasis.	Calcium<br>Tubulin<br>Cell motility
S100A8	*S100 calcium binding protein A8.* The protein is a member of the S100 protein family of proteins that are localized in the cytoplasm and/or nucleus and involved in regulation of several cellular processes such as cell cycle progression and differentiation. This protein possibly function in the inhibition of casein kinase and as a cytokine.	Casein kinase<br>Cytoplasm/nucleus<br>Cell cycle<br>Differentiation<br>Extracellular form
TLR2	*Toll like receptor 2.* The encoded receptor plays a fundamental role in pathogen recognition and activation of innate immunity. This receptor is a cell-surface protein that can form heterodimers with other TLR family members to recognize conserved molecules derived from microorganisms or endogenous proteins. Activation of TLRs leads to upregulation of signaling pathways. This receptor is also thought to promote apoptosis.	Pathogen receptor<br>Dimerization<br>Endogenous ligands
CD180	*CD180 molecule.* CD180 is a cell surface molecule with a short cytoplasmic tail. The extracellular part is associated with a molecule called MD-1 and form the cell surface receptor complex RP105/MD-1 that belongs to the family of pathogen receptors, Toll-like receptors (TLR). RP105/MD1 can work in concert with TLR4.	Pathogen receptor complex
ITPR1	*Inositol 1,4,5-trisphosphate receptor type 1.* This protein is an intracellular receptor for inositol 1,4,5-trisphosphate. Upon ligation the receptor mediates calcium release from the endoplasmic reticulum.	Recepor for inositol 1,4,5-trisphosphate<br>Calcium<br>Endoplasmic reticulum
ATP6V0D1	ATPase H+ transporting V0 subunit d1. This gene encodes a component of vacuolar ATPase (V-ATPase), a multisubunit enzyme that mediates acidification of intracellular organelles. V-ATPase dependent organelle acidification is necessary for such intracellular processes as protein sorting, zymogen activation and receptor-mediated endocytosis. V-ATPase is composed of a cytosolic V1 domain and a transmembrane V0 domain. The V1 domain consists of three A and three B subunits, two G subunits plus the C, D, E, F, and H subunits. The V1 domain contains the ATP catalytic site. The V0 domain consists of five different subunits: a, c, c', c", and d. This encoded protein is known as the D subunit.	V-ATPase<br>Organellar acidification

**RHO GTPases Activate NADPH Oxidases**<br>(Increased in primary AML cells)		
S100A8	See above	
S100A9	See above	
**Antimicrobial Peptides**<br>(Increased in primary AML cells)		
BP1	See above	
LYZ	See above	
S100A9	See above	
S100A8	See above	
TLR2	See above	
**Platelet function/(Integrin) signaling**<br>(decreased in primary AML cells)		
FGA	*Fibrinogen alpha chain.* This encoded alpha subunit of the coagulation factor fibrinogen; the encoded preproprotein is proteolytically processed by thrombin during the conversion of fibrinogen to fibrin.	Coagulation
FGB	*Fibrinogen beta chain.* The encoded protein is the beta component of fibrinogen, a blood-borne glycoprotein comprised of three pairs of nonidentical polypeptide chains. Various cleavage products of fibrinogen and fibrin regulate cell adhesion and spreading, display vasoconstrictor and chemotactic activities, and are mitogens for several cell types.	Coagulation<br>Antimicrobial<br>Cell adhesion/ Chemotaxis
FGG	*Fibrinogen gamma chain.* This protein is the gamma component of fibrinogen.	Coagulation<br>Antimicrobial<br>Cell adhesion/ Chemotaxis
FN1	*Fibronectin.* This glycoprotein is present in a soluble dimeric form in plasma, and in a dimeric or multimeric form at the cell surface and in extracellular matrix. The encoded preproprotein is proteolytically processed to generate the mature protein. Fibronectin is involved in cell adhesion and migration processes including wound healing, blood coagulation, host defense, and metastasis.	Adhesion<br>Cell migration
GP1B	*Glycoprotein Ib platelet subunit alpha.* Glycoprotein Ib is a platelet surface membrane glycoprotein composed of a heterodimer, an alpha chain and a beta chain, that is linked by disulfide bonds. The Gp Ib functions as a receptor for von Willebrand factor (VWF). The binding of the GP Ib-coagulation factor IX-V complex to VWF facilitates platelet adhesion to vascular subendothelium after vascular injury, and also initiates intracellular signaling that lead to enhanced platelet activation. This gene encodes the alpha subunit.	Platelet adhesion
ITGA2B	*Integrin subunit alpha 2b.* This member of the integrin alpha chain family of proteins is encoded as a preproprotein that is proteolytically processed to generate light and heavy chains that associate through disulfide linkages to form a subunit of the alpha-IIb/beta-3 integrin cell adhesion receptor.	Cell adhesion
**Volcano plot analysis**<br>(↑ means increased in AML cells, ↓means decreased in AML cells)		
AHNAK ↑	*AHNAK nucleoprotein.* The encoded protein is a large (700 kDa) structural scaffold protein that may play a role in diverse processes, including cancer cell migration and calcium channel regulation. A much shorter isoform/proteoform initiates a feedback loop that regulates alternative splicing of this gene.	Cell migration<br>Calcium regulation
HBA1 ↑	*Hemoglobin subunit alpha 1.* Two alpha chains plus two beta chains constitute HbA, which in normal adult life comprises about 97% of the total hemoglobin; alpha chains combine with delta chains to constitute HbA-2, which with HbF (fetal hemoglobin) makes up the remaining 3% of adult hemoglobin.	Erythroid differentiation

HBBB ↑	*Hemoglobin subunit beta.* The alpha (HBA) and beta (HBB) loci determine the structure of the 2 types of polypeptide chains in adult hemoglobin, Hb A. The normal adult hemoglobin tetramer consists of two alpha chains and two beta chains.	Erythroid differentiation
HIST1H1E ↑	*H1.4 linker histone, cluster member.* Histones are basic nuclear proteins responsible for the nucleosome structure of chromosomal fibers. Two molecules of each of the four core histones (H2A, H2B, H3, and H4) then form an octamer around which approximately 146 bp of DNA is wrapped in repeating units; this is called nucleosomes. The linker histone, H1, interacts with linker DNA between nucleosomes and functions in the compaction of chromatin into higher order structures. This gene encodes a replication-dependent histone that is a member of the histone H1 family.	Histone<br>Nucleosome
PLBD1 ↑	*Phospholipase B domain containing 1.* The protein is predicted to have phospholipase activity and is possibly involved in phospholipid catabolic process. It can be located in the extracellular space.	Lipid metabolism
ALDH1A1 ↓	*Aldehyde dehydrogenase 1 family member A1.* This protein belongs to the aldehyde dehydrogenase family. There are two major aldehyde dehydrogenase isozymes in the liver, cytosolic and mitochondrial, that are encoded by distinct genes. This gene encodes the cytosolic isozyme. Animal studies show that through its role in retinol metabolism, this gene may also be involved in the regulation of lipid metabolism.	Cytosolic enzyme<br>Lipid metabolism
APCS ↓	*Amyloid P component, serum.* This glycoprotein belongs to the pentraxin protein family. The binding of this protein to proteins in the pathological amyloid cross-beta fold suggests its possible role as a chaperone, and it is also thought to control the degradation of chromatin. It has been demonstrated that this protein binds to apoptotic cells at an early stage, which raises the possibility that it is involved in dealing with apoptotic cells in vivo.	Chaperon<br>Chromatin degradation
PHGDH ↓	*Phosphoglycerate dehydrogenase.* The encoded enzyme is involved in the early steps of L-serine synthesis. L-serine is required for D-serine and other amino acid synthesis. The enzyme requires NAD/NADH.	Amino acid metabolism<br>NAD/NADH
PRG2 ↓	*Proteoglycan 2, pro eosinophil major basic protein.* This protein is the predominant constituent of the crystalline core of the eosinophil granule. The proform of this protein can be present in serum where it forms a complex with several other proteins including angiotensinogen (AGT), and C3dg. It contains a peptide that displays potent antimicrobial activity against Gram-positive bacteria, Gram-negative bacteria, and fungi.	Antimirobial activity<br>Eosinophilic granules
TF ↓	*Transferrin.* This protein transports iron from the intestine, reticuloendothelial system, and liver parenchymal cells to all proliferating cells in the body. This protein may also be involved in removal of certain extracellular organic matters.	Iron metabolism

Table S7. Hierarchical clustering analysis of 121 differentially abundant proteins when comparing AML cells and normal CD34^{+} bone marrow cells; the protein subclassification into two main clusters as shown in Figure 5. Each main cluster could be further divided into two subclusters. The proteins are listed from the top of Figure 5 and downwards. The colors refer to the Reactome classification Neutrophil degranulation-Innate immune system, RHO GTPase activates NADPH oxidase, antimicrobial peptides and Integrin signaling/platelet activation. Protein names marked with grey shadow indicate proteins that were identified in the Volcano plot analysis Figure 4, light blue marking indicate proteins identified in the protein network analyses (Figure 4 lower left) and dark blue marking shows proteins identified in both in the Volcano and protein-protein interaction analyses (see Figure 3/4).

Proteins in the upper main cluster Platelet activation						Proteins in the lower main cluster Neutrophil degranulation					
Upper subcluster	Lower subcluster					Upper subcluster	Lower subcluster				
Protein name	Protein name	Neutrophil degranulation/innate immune system	RHO GTPases activate NADPH oxidases	Antimicrobial peptides	Platelet activation/function terms	Protein name	Protein name	Neutrophil degranulation/innate immune system	RHO GTPases activate NADPH oxidases	Antimicrobial peptides	Platelet activation/function terms
HSPB1	ITGA2B					PDPR	CBL				
FHL1	GP1BA					PBXIP1	SMAP2				
CD9	GP1BB					TRAF3IP3	ANXA2				
UHRF1	FGA					HIST1H1E	AHNAK				
CDK1	FGG					CA2	SULT1A1				
SMC4	FGB					HBB	APAF1				
PCNA	IGHA1					HBA1	DPYD				
SMC2	ALDH1A1					LGALS1	ATG7				
TOP2A	IGKV3-20						SH3BP1				
MKI67	JADE2						PRKCD				
LIG1	APOC3						APOBR				
MAD2L1	APCS						SERPINA3				
ROCK2	TTN						GRN				
CAD	HPX						LYZ				
PSMG2	FN1						MNDA				
RRM1	TMEM123						ATP6V0D1				
PTMA	TBC1D15						S100A11				
STMN1	CCNK						SIRPA				
DNPH1	PPP1R10						GLIPR2				
FKBP4	TF						S100A9				
ISYNA1	DENND5A						S100A8				
RCOR3	PIK3CB						PLBD1				
DUT	PRAME						CD180				
GRWD1	CLC						ITGAL				
TRMT61A	EPX						TLR2				
ADH7	FKBP11						SCPEP1				
TMEM14C	PRG2						ITGAX				
PHGDH	MZB1						IFI30				
SACS	AKR1C3						CAPN2				
DBN1	H1F0						KCTD12				
SCRN1	ALDH7A1						NCF1				
CMBL	MSI2						ITPR1				
ACY1	HMGN5						ATM				
	REXO2						GNS				
	CD59						SYNE3				
	HDAC7						TSPO				
	TFRC						CD97				
	BPI						PLEC				
	PRTN3						M<VP				
	HP										
	MSL1										

Table S8. Clinical and biological characteristics of the 50 AML patients included in the study. Patients are listed according to the clustering analysis presented in Figure 5 (listed from left to right, see the top of the figure) in the article, i.e. the upper part of the table represents the left 22 patients in the cluster analysis and the lower part the right subset of 28 patients. The table presents the patient identity, gender (M, male; F, female), patients with secondary AML (MDS, myelodysplastic syndrome; Chemo, previous chemotherapy), karyotype and molecular genetic abnormalities. (M, mutated; ITD-internal tandem duplication, TKD, tyrosine kinase domain) and time to/course after relapse (CR1/CR2, first/second complete hematological remission; HSCT, hematopoietic stem cell transplantation; NRM, non-relapse mortality; RELFREE, long-term relapse-free survival; RESISTANT, primary resistance). The survivors had an observation time of at least seven years. The yellow color in the patient identity column indicates that these patients belonged to the subset of 10 exceptional patients that clustered together with the normal CD34^{+} bone marrow cells in the clustering analysis based on the 16 differentially abundant proteins involved in the regulation of iron metabolism/ferroptosis (Figure 6).

Patient	Gender	Age (years)	Secondary	FAB classification	CD34	Karyotype	*NPM1* Insertion	*FLT3*-ITD	*FLT3*-TKD	Time when allo-HSCT	Long-term survival	Clinical course after conventional chemotherapy
P30	M	67		M0	+	45-46,XY,-5,+dic(1;5)(1p1?;5q13)		M				RELAPSE
P33	F	56		M1	+	Nomal						RESISTANT
P9	M	46		M1	nt	Normal	M					RELAPSE
P6	F	66		M1	-	Normal	M					RELAPSE
P21	F	48		M0	+	nt	nt	nt	nt			RELAPSE
P40	F	63		M1	+	dup(11)(q23.3q23.3)			M			RELAPSE
P15	F	67		M0	+	+6,+21,inc[4]/46, XX[17]						RELAPSE
P10	M	57		M4	-	Normal					NRM	RELFREE
P3	M	56		M1	+	46,XY,inv(1)(p22?p34?) t(2;10?)(q33?;q22?),t(9;22)(q34;q11)	nt		nt		NRM	RELFREE
P20	F	55		M0	+	Normal		M				RELAPSE
P17	M	60		M4	+	del(9)(q13q33)		M				RELAPSE
P32	M	62		M1	+	Nt						RELAPSE
P26	M	53		M0	+	Complex				CR2	+	RELAPSE
P8	M	24		M2	+	Complex				CR1		RELFREE
P48	M	68		M1	+	Normal						RELAPSE
P18	F	59	MDS	M5	-	Normal	M	M				RELAPSE
P13	F	45	Chemo	M4	-	Normal	M				+	RELFREE
P35	F	80		M2	+	Complex			M			RESISTANT
P2	M	29		M4	+	Normal	M	M				RELAPSE
P45	F	61	MDS	M5	+	Normal		M			NRM	RELAPSE
P16	F	58		M2	+	nt						RELAPSE
P52	M	62		M4	+	+8[12]/46,XY[13]						RELAPSE
P4	M	60		M0/1	+	nt						RELAPSE
P24	M	53		M4	-	Normal	M				+	RELFREE
P50	F	36		M4	-	Normal	nt	nt	nt			RELAPSE
P49	M	54	Chemo	M5	-	Normal	M				+	RELFREE
P14	F	42		M5	-	Normal	M				+	RELFREE
P44	M	65		M5	nt	Complex	M				+	RELFREE
P46	F	60		M5	-	Normal	M	M			+	RELFREE
P34	M	48		M5	-	Normal	M	M	M			RELAPSE
P22	M	41		M4	nt	Normal						RELAPSE
P47	F	41		M4	-	Normal	M				+	RELFREE

P31	F	36	Chemo	M5	-	t(9;11)(p21;q23)					+	**RELFREE**
P7	F	61		M5	-	t(2;3)(q37;q21),t(2;4;10)(q13;q21)q21)<br>der(11q),19q+						**RESISTANT**
P23	M	46		M4	nt	Normal	M	M				**RELAPSE**
P43	M	48		M4	+	inv(16)(p13q22)					+	**RELFREE**
P36	M	36		M4	+	inv(16)(p13q22)					+	**REL_FREE**
P53	M	48		M4	-	Normal	M				+	**RELFREE**
P51	F	32		M5	-	del(5)(q31q34)	M			CR2	+	**RELAPSE**
P29	M	58		M5	+	Normal					+	**RELFREE**
P41	F	51		M1	+	Nt		nt	nt			**RELAPSE**
P19	M	35		M2	+	Normal				CR2	+	**RELAPSE**
P39	F	29		M2	+	Normal		M	M	CR2	+	**RELFREE**
P38	M	20		M2	-	Normal		M		CR2		**RELAPSE**
P1	M	42		M2	-	Normal		M		CR1	+	**RELFREE**
P5	M	27		M2	+	Normal	nt	nt	nt	CR2	+	**RELAPSE**
P37	F	57		M4	+	46,XX,inv(16)(13;q22)[5]<br>46,idem,t(X;6)(p22;p12)[13]/46,XX[2]						**RELAPSE**
P12	F	46		M1	+	inv(16)(p13q22)				CR2	+	**RELAPSE**
P11	F	18		M4	+	inv(16)(q13q22)				CR2	+	**RELAPSE**
P42	M	43		M5	+	inv(16)					+	**RELFREE**

Table S9. Clinical and biological characteristics of 50 AML patients included in the study; a comparison of two patient subsets identified by unsupervised hierarchical clustering analysis (Figure 5). Patients are classified according to the clustering analysis presented in Figure 5 and Table S8 as described in the article. The table compares the two patient subsets with regard to sex, bone marrow blasts (percent of nucleated cells), peripheral blood blast count (x 10^9/L), FAB classification/morphology of the leukemic cells, CD34 expression of the AML cells, karyotype and molecular genetic abnormalities (FLT3-ITD, NPM1-Ins) and resistance/survival.

**Parameter**	**The left patient subset (22 patients)**	**The right patient subset (28 patients)**	**p-value**
Sex (male/female)	11/11	16/12	0.7759
**Age (years, median and range)**	**Median 61 (range 29-80)** **IQR 63.75 - 51.75 = 12**	**Median 45 (range 18-65)** **IQR 53.75 - 36 = 17.75**	**0.00076**
Bone marrow blasts at diagnosis (percentage)	86(25-99)	77(30-97)	0.09497
Circulating blasts at diagnosis (x 10^9/L)	23.1(6.2-114)	37.6(3.2-182)	0.2098
**FAB classification M0/M1 versus others**	**12 versus 10**	**2 versus 26**	**0.0003**
FAB classification M0 versus others	4 versus 18	0 versus 28	0.0318
**$CD34^+$ AML cells >20%**	**17 versus 4**	**12 versus 13**	**0.0307**
**Karyotype favorable versus others**	**0 versus 20**	**6 versus 21**	**0.0312**
**Karyotype favorable plus normal versus others**	**10 versus 10**	**22 versus 5**	**0.0299**
Flt3-ITD	6 versus 15	6 versus 20	0.7438
NPM1-Ins	5 versus 17	10 versus 18	0.3673
**Primary resistant/relapse after initial therapy**	**18 versus 4**	**14 versus 14**	**0.0365**
**Long-term AML-free survival**	**4 versus 17**	**19 versus 10**	**0.0016**

Table S10. Protein phosphorylation of primary human AML cells, the identification of protein phosphorylation sites that (i) are localized on a subset (a total of 38 proteins) of the 121 proteins that showed statistically significant differential expression and at least a 2-fold difference when comparing primary AML cells and normal CD34^{+} bone marrow cells; and (ii) in addition a statistically significant difference for at least one phosphosite when comparing the two patient subsets identified in the unsupervised hierarchical clustering analysis presented in Figure 5 (i.e. 23/28 right versus 18/22 left patients). The first criteria identified 174 phosphosites on 38 differentially abundant proteins, after applying the additional second criteria we identified 53 phosphosites on 16 differentially abundant proteins that are listed in the table. The table presents the gene name, identified phosphosite(s), protein name, p-value and the fold change (FC) when comparing the two patient subsets identified in Figure 5 in the article (the larger main subset including 28 patients versus the smaller left subset including 22 patients). The comparison of these two subsets was based on cells derived from 23 unselected patients from the 28 patients in the right subset and 18 of the 22 patients in the left cluster. Red color indicates that the phosphorylation was generally higher in the right subset whereas blue color indicates higher phosphorylation in the left subset.

Gene name	Phosphosite	Protein name	Welch t-test p-value	FC 28/22
AHNAK	AHNAK_S5110	Neuroblast differentiation-associated protein AHNAK	0.0045	0.838
	AHNAK_S5749		0.0276	1.034
	AHNAK_S4986		0.0137	1.698
	AHNAK_S5830		1.3099E-05	1.591
	AHNAK_S3411		5.27279E-05	1.511
	AHNAK_S5720		0.0395	2.293
	AHNAK_S5769		0.0122	1.764
	AHNAK_S93		0.0005	0.747
	AHNAK_S5731		0.0032	1.098
	AHNAK_S5841		0.0011	0.530
	AHNAK_S5448		0.0058	1.492
	AHNAK_S2397		0.0016	1.427
	AHNAK_S570		0.0008	1.277
	AHNAK_S135		2.77988E-05	1.529
	AHNAK_S210		0.0089	0.953
	AHNAK_T490		0.0218	1.117
	AHNAK_S5749		0.0001	1.042
	AHNAK_S5752		2.19296E-05	1.256
	AHNAK_S5780		5.19477E-05	1.050
	AHNAK_S5782		0.0018	1.050
	AHNAK_S210		0.0032	1.388
	AHNAK_S216		0.0170	1.183
	AHNAK_S5752		0.0471	1.166
	AHNAK_S5745		0.0324	1.061
	AHNAK_S5746		0.0183	1.027
ANXA2	ANXA2_S26	Annexin A2;Putative annexin A2-like protein	0.0067	1.178
APOBR	APOBR_S891	Apolipoprotein B receptor	0.0277	1.202
	APOBR_S175		0.0010	2.206
	APOBR_S162		0.0434	0.969
DBN1	DBN1_S142	Drebrin	8.09099E-08	-2,532
DUT	DUT_S11	Deoxyuridine 5'-triphosphate nucleotidohydrolase, mitochondrial	0.0387	-1.119
GP1BB	GP1BB_S191	Platelet glycoprotein Ib beta chain	0.0070	-1.592
ITPR1	ITPR1_S1716	Inositol 1,4,5-trisphosphate receptor type 1	0.0455	0.2590
KCTD12	KCTD12_S187	BTB/POZ domain-containing protein KCTD12	0.0125	1.3189
MSL1	MSL1_S205	Male-specific lethal 1 homolog	0.0187	-0.485

PBXIP1	PBXIP1_S43	Pre-B-cell leukemia transcription factor-interacting protein 1	0.0048	-0.949
PLEC	PLEC_S4386	Plectin	0.0131	1.204
	PLEC_T4030		0.0249	0.704
	PLEC_S4386		0.0007	1.464
	PLEC_S4389		0.0010	1.418
	PLEC_S4396		2.54364E-05	1.373
	PLEC_S4622		0.0012	1.401
	PLEC_S4626		0.0010	1.379
PRKCD	PRKCD_S645	Protein kinase C delta type	0.0052	1.116
	PRKCD_S304		0.0031	1.833
RCOR3	RCOR3_S372	REST corepressor 3	0.0072	-0.892
	RCOR3_S375		0.0072	-0.892
	RCOR3_T376		0.0104	-0.872
SMC4	SMC4_S27	Structural maintenance of chromosomes protein 4	0.0192	0.535
STMN1	STMN1_S25	Stathmin	0.0166	0.577
	STMN1_S38		0.0136	-.1388
TOP2A	TOP2A_S1374	DNA topoisomerase 2-alpha	0.0218	1.158
	TOP2A_S1377		0.0286	0.956

Table S11. Differentially abundant proteins showing additional differences in protein phosphorylation. We compared AML cells derived from 50 patients and normal $CD34^+$ bone marrow cells derived from eight healthy individuals; an overview of identified proteins after analysis based on Welch's t-test with Benjamini correction and fold-change significance (z-score test). The statistical analysis was based on those proteins having at least 70% valid ratios in both groups. The table presents those 16 differentially abundant proteins (i) whose level differed significantly between AML cells and normal $CD34^+$ bone marrow cells; (ii) showing at least a 2-fold difference and (iii) additional significant differences in at least one phosphorylation site.

GENE NAMES	PROTEIN NAMES	KEY WORDS
AHNAK	*AHNAK nucleoprotein.* The encoded protein is a large (700 kDa) structural scaffold protein that may play a role in diverse processes, including cancer cell migration and calcium channel regulation. A much shorter isoform/proteoform initiates a feedback loop that regulates alternative splicing of this gene.	Cell migration<br>Calcium regulation
ANXA2	*Annexin A2;Putative annexin A2-like protein.* This encoded member of the calcium-dependent phospholipid-binding annexin protein family is involved in regulation of cellular growth and in signal transduction pathways. It can function as an autocrine factor. Multiple alternatively spliced transcript variants encoding different isoforms/proteoforms have been detected. Annexin A2 expression correlates with chemoresistance in various cancer forms.	Cell growth<br>Intracellular signaling<br>Autocrine factor<br>chemoresistance
APOBR	*Apolipoprotein B receptor.* The apolipoprotein B48 receptor is a macrophage receptor that binds to an apolipoprotein of dietary triglyceride-rich lipoproteins. This receptor may provide essential lipids, lipid-soluble vitamins and other nutrients.	Nutrition
DBN1	*Drebrin.* This is a cytoplasmic actin-binding protein. It is a member of the drebrin protein family. At least two alternative splice variants encoding different protein isoforms/proteoforms have been described.	Actin binding
DUT	*Deoxyuridine 5'-triphosphate nucleotidohydrolase, mitochondrial.* The encoded protein is an essential enzyme of nucleotide metabolism. It forms a homotetrameric enzyme that hydrolyzes dUTP to dUMP and pyrophosphate; this reaction provides a precursor (dUMP) for synthesis of thymine nucleotides needed for DNA replication and limits intracellular pools of dUTP. High dUTP levels lead to increased incorporation of uracil into DNA, which induces extensive excision repair mediated by uracil glycosylase, a self-defeating process resulting in removal and reincorporation of dUTP, DNA fragmentation and cell death. Alternative splicing leads to different isoforms/proteoforms that localize to either the mitochondrion or nucleus.	Nucleotide metabolism<br>Viability/cell death
GP1BB	*Platelet glycoprotein Ib beta chain.* The encoded protein is a part of a heterodimeric transmembrane protein consisting of a disulfide-linked 140 kD alpha chain and 22 kD beta chain. It is part of the receptor for von Willebrand factor, and GPIb beta contributes to surface expression of the receptor and participates in transmembrane signaling through phosphorylation of its intracellular domain. The protein can also be abundant in nonhematopoietic tissues.	von Willebrand factor<br>Intracellular signaling
ITPR1	*Inositol 1,4,5-trisphosphate receptor type 1.* This encoded intracellular receptor for inositol 1,4,5-trisphosphate mediates calcium release from the endoplasmic reticulum after ligation. Multiple transcript variants have been identified.	Intracellular signaling<br>Calcium release
KCTD12	*BTB/POZ domain-containing protein KCTD12.* The encoded plasma membrane protein is predicted to be involved in regulation of G protein-coupled receptor signaling pathway.	Intracellular signaling
MSL1	*Male-specific lethal 1 homolog.* The encoded protein has protein-macromolecule adaptor activity. It is involved in DNA-templated transcription and is located in nucleoplasm. Part of MSL complex.	Transcription
PBXIP1	*Pre-B-cell leukemia transcription factor-interacting protein 1.* The encoded protein interacts with the PBX1 homeodomain protein and inhibits its transcriptional activation by preventing its binding to DNA. It is primarily cytosolic but can shuttle to the nucleus, and it can promote the proliferation of various malignant tumor cells cancer. Several transcript variants encoding different isoforms/proteoforms have been detected.	Transcription<br>Carcinogenesis
PLEC	Plectin. Plectin is a member of a family of structurally and in part functionally related proteins termed plakins or cytolinkers that are able to interlink different elements of the cytoskeleton and in addition serve as scaffolding platforms for the assembly, positioning, and regulation of signaling complexes. Plectin is abundant as several protein isoforms/proteoforms in a wide range of cell types. The protein contains an actin binding domain. Isoforms/proteoforms lacking the central rod domain encoded by exon 31 have been detected in humans, and certain isoforms/proteoforms are located to distinct subcellular sites. It appears that each cell type contains a unique set of plectin isoforms/proteoforms.	Cytoskeleton<br>Intracellular signaling

PRKCD	*Protein kinase C delta type.* The encoded protein is a member of the protein kinase C family of serine- and threonine-specific protein kinases. It is activated by diacylglycerol and is both a tumor suppressor, a positive regulator of cell cycle progression and a regulator of apoptosis.	Tumor suppressor<br>Cell cycle<br>Apoptosis
RCOR3	*REST corepressor 3.* The encoded protein is predicted to have enzyme binding activity and transcription corepressor activity, i.e. being involved in negative regulation of DNA-templated transcription and regulation of transcription by RNA polymerase II. It is located in cytosol and nucleoplasm.	Transcription
SMC4	Structural maintenance of chromosomes protein 4. This gene belongs to the 'structural maintenance of chromosomes' (SMC) gene family, whose members play a role in changes in chromosome structure during mitotic segregation. The encoded protein is probably a subunit of the 13S condensin complex, which is involved in chromosome condensation.	Mitosis
STMN1	Stathmin. The encoded protein belongs to the stathmin family and is a cytosolic phosphoprotein proposed to function as an intracellular relay integrating regulatory signals of the cellular environment. It is involved in the regulation of the microtubule filament system by destabilizing microtubules. Multiple transcript variants encoding different isoform/proteoforms s have been detected.	Intracellular signaling<br>Microtubules
TOP2A	DNA topoisomerase 2-alpha. This gene encodes a DNA topoisomerase, an enzyme that controls and alters the topologic states of DNA during transcription. This nuclear enzyme is involved in processes such as chromosome condensation, chromatid separation, and the relief of torsional stress that occurs during DNA transcription and replication. It catalyzes the transient breaking and rejoining of two strands of duplex DNA which allows the strands to pass through one another, thus altering the topology of DNA. The gene encoding this enzyme functions as the target for several anticancer agents and a variety of mutations in this gene have been associated with the development of drug resistance.	Transription<br>Chemoresistance

Table S12. Proteins involved in the regulation of iron homeostasis/metabolism and/or ferroptosis and showing differential expression when comparing primary AML cells and normal CD34$^+$ bone marrow cells. The proteins are listed according to the clustering analysis presented in Figure 5. These proteins showed differential expression when comparing AML cells derived from 50 patients and normal CD34$^+$ bone marrow cells derived from eight healthy individuals. They were identified after statistical analysis based on Welch's t-test with Benjamini correction and fold-change significance (z-score test, $p<0.05$). The statistical analysis was based on those proteins having at least 70% valid ratios in both groups. The information is based on the Gene database (accessed December 05, 2024) and selected references.

GENE NAME	PROTEIN NAME AND FUNCTION	KEY WORDS
**Upper main cluster Figure 5** (Iron absorbtion and ubiquitin)		
TFRC	*Transferrin receptor*. This gene encodes a cell surface receptor necessary for cellular iron uptake by the process of receptor-mediated endocytosis.	Endocytosis<br>Iron uptake
RPS27A	*Ribosomal protein S27a.* Ubiquitin has a major role in targeting cellular proteins for degradation by the 26S proteasome. This gene encodes a fusion protein consisting of ubiquitin at the N terminus and ribosomal protein S27a at the C terminus. When abundant in yeast, the protein is post-translationally processed, generating free ubiquitin monomer and ribosomal protein S27a. Ribosomal protein S27a is a component of the 40S ribosomal subunit and is located in the cytoplasm.	Ribosome<br>Ubiquitin<br>Cytoplasm
TF	*Transferrin.* The encoded glycoprotein has an approximate molecular weight of 76.5 kDa and is thought to have been created as a result of an ancient gene duplication event that led to generation of homologous C and N-terminal domains each of which binds one ion of ferric iron. The function of this protein is to transport iron from the intestine, reticuloendothelial system, and liver parenchymal cells to all proliferating cells in the body. This protein may also have a physiologic role as granulocyte/pollen-binding protein (GPBP) involved in the removal of certain organic matters and allergens from serum.	Iron transport
**Lower main cluster, upper subcluster** (Oxidoreductation/glutathion, ubiquitination, iron absorption regulation)		
GLRX3	*Glutaredoxin 3.* This gene encodes a member of the glutaredoxin family, i.e. oxidoreductase (i.e. electron transfer) enzymes that reduce a variety of substrates using glutathione as a cofactor. The protein binds to and modulates the function of protein kinase C theta. It may also inhibit apoptosis and play a role in cellular growth, and the expression of this gene may be a marker for cancer. It is regarded as a regulator of iron homeostasis [89,90]. Alternatively spliced transcript variants have been observed for this gene.	Redox balance<br>Glutaredoxin<br>Glutathion<br>Antiapoptotic
CAND1	*Cullin associated and neddylation dissociated 1.* The encoded protein is an essential regulator of Cullin-RING (Ring box 1) ubiquitin ligases, which are in involved in ubiquitinylation of proteins degraded by the Ub proteasome system. The encoded protein binds to unneddylated cullin-RING box protein complexes and acts as an inhibitor of cullin neddylation and of Skp1, cullin, and F box ubiquitin ligase complex assembly and activity. In mammalian cell culture, this protein predominantly localizes to the cytoplasm.	Cytoplasm<br>Ubiquitin-dependent protein catabolism
ACO1	*Aconitase 1.* The encoded protein is a bifunctional, cytosolic protein that functions as an essential enzyme in the tricarbonic acid cycle and interacts with mRNA to control the levels of iron inside cells. When cellular iron levels are high, this protein binds to a 4Fe-4S cluster and functions as an aconitase. Aconitases are iron-sulfur proteins that function to catalyze the conversion of citrate to isocitrate. When cellular iron levels are low, the protein binds to iron-responsive elements (IREs), which are stem-loop structures found in the 5' UTR of ferritin mRNA, and in the 3' UTR of transferrin receptor mRNA. When the protein binds to IRE, it results in repression of translation of ferritin mRNA, and inhibition of degradation of the otherwise rapidly degraded transferrin receptor mRNA. Alternative splicing results in multiple transcript variants	Iron absorption regulation<br>Transferrin receptor<br>Transferrin<br>Ferritin<br>RNA
CUL1	*Cullin 1.* The encoded protein has ubiquitin ligase complex scaffold activity and ubiquitin protein ligase binding activity. It is involved in proteasomal ubiquitin-dependent protein catabolic process, positive regulation of canonical NF-kappaB signal transduction and protein K48-linked ubiquitination. The protein is located in the plasma membrane and is a part of ubiquitin ligase complexes, and the crosstalk between iron and the protein ubiquitination system is important in the regulation of cellular iron homeostasis [91,92]).	Plasma membrane<br>Ubiquitin-dependent protein catabolism<br>NF-kappaB signaling

PCBP1	Poly(rC) binding protein 1. This intronless gene is thought to have been generated by retrotransposition of a fully processed PCBP-2 mRNA. The encoded multifunctional protein along with PCBP-2 and hnRNPK corresponds to the major cellular poly(rC)-binding proteins. It may be involved in RNA binding. It has also been implicated in translational control. The encoded protein is also suggested to play a part in formation of a sequence-specific alpha-globin mRNP complex which is associated with alpha-globin mRNA stability.	RNA binding<br>Translation
NEDD8	*NEDD8 ubiquitin like modifier*. The encoded protein has ubiquitin protein ligase binding activity, it acts upstream of or within protein neddylation and is located in cytosol and nucleoplasm [92-94].	Ubiquitin
	**Lower main cluster, lower subcluster**<br>(Fatty acid metabolism, endosomal acidification and autophagy)	
ACSL1	*Acyl-CoA synthetase long chain family member 1*. The encoded protein is an isozyme of the long-chain fatty-acid-coenzyme A ligase family. Although differing in substrate specificity, subcellular localization, and tissue distribution, all isozymes of this family convert free long-chain fatty acids into fatty acyl-CoA esters, and thereby play a key role in lipid biosynthesis and fatty acid degradation. Several transcript variants encoding different isoforms/proteoforms have been found for this gene.	Fatty acid metabolism
ATP6V0D1	*ATPase H+ transporting V0 subunit d1*. This gene encodes a component of vacuolar ATPase (V-ATPase), a multisubunit enzyme that mediates acidification of intracellular organelles. V-ATPase dependent organelle acidification is necessary for such intracellular processes as protein sorting, zymogen activation, receptor-mediated endocytosis, and vesicle proton gradient generation. V-ATPase is composed of a cytosolic V1 domain and a transmembrane V0 domain. The V1 domain consists of three A, three B, and two G subunits, as well as a C, D, E, F, and H subunit. The V1 domain contains the ATP catalytic site. The V0 domain consists of five different subunits: a, c, c', c", and d. Additional isoforms/proteoforms of many of the V1 and V0 subunit proteins are encoded by multiple genes or alternatively spliced transcript variants. This encoded protein is known as the D subunit and is found ubiquitously.	V-ATPase
ATP6V1A	*ATPase H+ transporting V1 subunit A*. This gene also encodes a component of vacuolar ATPase (V-ATPase). The encoded protein is one of two V1 domain A subunit isoforms/proteoforms and is found in all tissues.	V-ATPase
ATP6V1B2	*ATPase H+ transporting V1 subunit B2*. This gene encodes a component of vacuolar ATPase (V-ATPase). The protein is one of two V1 domain B subunit isoforms/proteoforms and is the only B isoform/proteoform highly abundant in osteoclasts.	V-ATPase
ATP6V1G1	*ATPase H+ transporting V1 subunit G1*. This gene encodes a component of vacuolar ATPase (V-ATPase). The protein is one of three V1 domain G subunit proteins.	V-ATPase
ATP6V1E1	*ATPase H+ transporting V1 subunit E1*. This gene encodes a component of vacuolar ATPase (V-ATPase) The gene encodes alternate transcriptional splice variants, encoding different V1 domain E subunit isoforms/proteoforms.	V-ATPase
ATG7	*Autophagy related 7,* This encoded E1-like activating enzyme is essential for autophagy and cytoplasmic to vacuole transport. It is also thought to modulate p53-dependent cell cycle pathways during prolonged metabolic stress. It has been associated with multiple functions, including mitophagy and hematopoietic stem cell maintenance. Alternative splicing results in multiple transcript variants.	Autophagy/mitophagy<br>Hematopoietic stem cell maintenance<br>Cell cycle regulation

Table S13. Clinical and biological characteristics of the 50 AML patients included in the study; patient subclassification in an unsupervised hirerachical clustering analysis based on 16 differentially abundant proteins involved in regulation of iron metabolism and/or ferroptosis (Figure 6) (listed from left to right, see the top of the figure). The separation between subclusters is marked with light green and between main clusters dark green. The 10 exceptional patients clustering together with the normal CD34^{+} bone marrow cells are marked with grey. The table presents the patient identity, gender (M, male; F, female), patients with secondary AML (MDS, myelodysplastic syndrome; Chemo, previous chemotherapy), karyotype and molecular genetic abnormalities. (M, mutated; ITD-internal tandem duplication, TKD, tyrosine kinase domain) and time to/course after relapse (CR1/CR2, first/second complete hematological remission; HSCT, hematopoietic stem cell transplantation; NRM, non-relapse mortality; RELFREE, long-term relapse-free survival; RESISTANT, primary resistance). The survivors had an observation time of at least seven years.

Patient	Gender	Age (years)	Secondary	FAB classification	CD34	Karyotype	*NPM1* Insertion	*FLT3*-ITD	*FLT3*-TKD	Time when allo-HSCT	Long-term survival	Clinical course after conventional chemotherapy
P3	M	56		M1	+	46,XY,inv(1)(p22?p34?) t(2;10?)(q33?;q22?),t(9;22)(q34;q11)	nt		nt		NRM	RELFREE
P26	M	53		M0	+	Complex				CR2	+	RELAPSE
P5	M	27		M2	+	Normal	nt	nt	nt	CR2	+	RELAPSE
P40	F	63		M1	+	dup(11)(q23.3q23.3)			M			RELAPSE
P42	M	43		M5	+	inv(16)					+	RELFREE
P13	F	45	Chemo	M4	-	Normal	M				+	RELFREE
P17	M	60		M4	+	del(9)(q13q33)		M				RELAPSE
P29	M	58		M5	+	Normal					+	RELFREE
P12	F	46		M1	+	inv(16)(p13q22)				CR2	+	RELAPSE
P21	F	48		M0	+	Nt	nt	nt	nt			RELAPSE
P15	F	67		M0	+	+6,+21,inc[4]/46, XX[17]						RELAPSE
P41	F	51		M1	+	Nt		nt	nt			RELAPSE
P52	M	62		M4	+	+8[12]/46,XY[13]						RELAPSE
P19	M	35		M2	+	Normal				CR2	+	RELAPSE
P8	M	24		M2	+	Complex				CR1		RELFREE
P9	M	46		M1	Nt	Normal	M					RELAPSE
P20	F	55		M0	+	Normal		M				RELAPSE
P10	M	57		M4	-	Normal					NRM	RELFREE
P37	F	57		M4	+	46,XX,inv(16)(13;q22)[5] 46,idem,t(X;6)(p22;p12)[13]/46,XX[2]						RELAPSE
P11	F	18		M4	+	inv(16)(q13q22)				CR2	+	RELAPSE
P38	M	20		M2	-	Normal		M		CR2		RELAPSE
P1	M	42		M2	-	Normal		M		CR1	+	RELFREE
P47	F	41		M4	-	Normal	M				+	RELFREE
P7	F	61		M5	-	t(2;3)(q37;q21),t(2;4;10)(q13;q21)q21) der(11q),19q+						RESISTANT
P36	M	36		M4	+	inv(16)(p13q22)					+	REL_FREE
P22	M	41		M4	nt	Normal						RELAPSE
P31	F	36	Chemo	M5	-	t(9;11)(p21;q23)					+	RELFREE
P39	F	29		M2	+	Normal		M	M	CR2	+	RELFREE
P34	M	48		M5	-	Normal	M	M	M			RELAPSE
P24	M	53		M4	-	Normal	M				+	RELFREE
P46	F	60		M5	-	Normal	M	M			+	RELFREE

P44	M	65		M5	nt	Complex	M				+	RELFREE
P49	M	54	Chemo	M5	-	Normal	M				+	RELFREE
P4	M	60		M0/1	+	Nt						RELAPSE
P14	F	42		M5	-	Normal	M				+	RELFREE
P50	F	36		M4	-	Normal	nt	nt	nt			RELAPSE
P23	M	46		M4	nt	Normal	M	M				RELAPSE
P53	M	48		M4	-	Normal	M				+	RELFREE
P43	M	48		M4	+	inv(16)(p13q22)					+	RELFREE
P51	F	32		M5	-	del(5)(q31q34)	M			CR2	+	RELAPSE
P33	F	56		M1	+	Nomal						RESISTANT
P6	F	66		M1	-	Normal	M					RELAPSE
P35	F	80		M2	+	Complex			M			RESISTANT
P18	F	59	MDS	M5	-	Normal	M	M				RELAPSE
P48	M	68		M1	+	Normal						RELAPSE
P30	M	67		M0	+	45-46,XY,-5,+dic(1;5)(1p1?;5q13)		M				RELAPSE
P32	M	62		M1	+	nt						RELAPSE
P16	F	58		M2	+	nt						RELAPSE
P2	M	29		M4	+	Normal	M	M				RELAPSE
P45	F	61	MDS	M5	+	Normal		M				RELAPSE
